# Death and Resurrection of the Human *IRGM* Gene

**DOI:** 10.1371/journal.pgen.1000403

**Published:** 2009-03-06

**Authors:** Cemalettin Bekpen, Tomas Marques-Bonet, Can Alkan, Francesca Antonacci, Maria Bruna Leogrande, Mario Ventura, Jeffrey M. Kidd, Priscillia Siswara, Jonathan C. Howard, Evan E. Eichler

**Affiliations:** 1 Department of Genome Sciences, University of Washington, Seattle, Washington, United States of America; 2 Howard Hughes Medical Institute, Seattle, Washington, United States of America; 3 Institut de Biologia Evolutiva (UPF-CSIC), Barcelona, Spain; 4 Universita' degli Studi di Bari, Bari, Italy; 5 Institute of Genetics, University of Cologne, Cologne, Germany; University of Aarhus, Denmark

## Abstract

Immunity-related GTPases (*IRG*) play an important role in defense against intracellular pathogens. One member of this gene family in humans, *IRGM*, has been recently implicated as a risk factor for Crohn's disease. We analyzed the detailed structure of this gene family among primates and showed that most of the *IRG* gene cluster was deleted early in primate evolution, after the divergence of the anthropoids from prosimians ( about 50 million years ago). Comparative sequence analysis of New World and Old World monkey species shows that the single-copy *IRGM* gene became pseudogenized as a result of an Alu retrotransposition event in the anthropoid common ancestor that disrupted the open reading frame (ORF). We find that the ORF was reestablished as a part of a polymorphic stop codon in the common ancestor of humans and great apes. Expression analysis suggests that this change occurred in conjunction with the insertion of an endogenous retrovirus, which altered the transcription initiation, splicing, and expression profile of *IRGM*. These data argue that the gene became pseudogenized and was then resurrected through a series of complex structural events and suggest remarkable functional plasticity where alleles experience diverse evolutionary pressures over time. Such dynamism in structure and evolution may be critical for a gene family locked in an arms race with an ever-changing repertoire of intracellular parasites.

## Author Summary

The *IRG* gene family plays an important role in defense against intracellular bacteria, and genome-wide association studies have implicated structural variants of the single-copy human *IRGM* locus as a risk factor for Crohn's disease. We reconstruct the evolutionary history of this region among primates and show that the ancestral tandem gene family contracted to a single pseudogene within the ancestral lineage of apes and monkeys. Phylogenetic analyses support a model where the gene has been “dead” for at least 25 million years of human primate evolution but whose ORF became restored in all human and great ape lineages. We suggest that the rebirth or restoration of the gene coincided with the insertion of an endogenous retrovirus, which now serves as the functional promoter driving human gene expression. We suggest that either the gene is not functional in humans or this represents one of the first documented examples of gene death and rebirth.

## Introduction

Immunity Related GTPases (*IRG*), a family of genes induced by interferons, are one of the strongest resistance systems to intracellular pathogens [1]–[4]. The *IRGM* gene has been shown to have a role in the autophagy-targeted destruction of *Mycobacterium bovis BCG* [5]. Recently, whole genome association studies have shown that specific *IRGM* haplotypes associate with increased risk for Crohn's disease [6],[7]. The *IRG* gene family exists as multiple copies (3–21) in most mammalian species but has been reduced to two copies, *IRGC* and a truncated gene *IRGM*, in humans [8]. Analysis of mammalian genomes (dog, rat and mouse) has shown that all *IRG* genes except *IRGC* are organized in tandem gene clusters mapping to mouse chromosomes 11 and 18 (both syntenic to human chromosome 5) [8]. A comparison of the mouse and human genomes identified 21 genes in mouse but only a single syntenic truncated *IRGM* copy and *IRGC* in human [8]. We investigated the copy number and sequence organization of the *IRG* gene family in multiple nonhuman primate species in order to reconstruct the evolutionary history of this locus.

## Results

**Figure 1:**
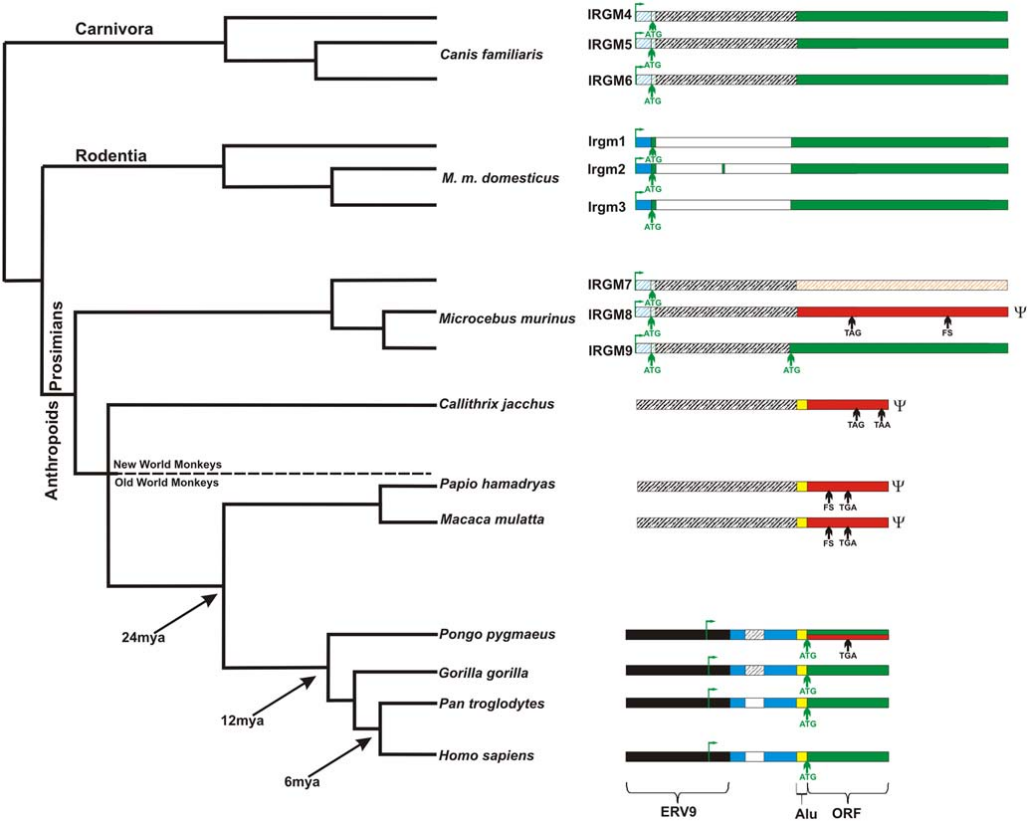
Comparative structure of *IRGM* loci. The structures of the *IRGM* loci are shown in the context of a generally-accepted primate phylogenetic tree. ORF, ERV9, intronic sequence, Alu sequence, and 5′ untranslated region (UTR) depicted in green, black, white, yellow and blue colors respectively. A red color denotes pseudogenes based on the accumulation of deleterious mutations in the ORF. Shaded orange color indicates an atypical GTPase because of mutations leading to the loss of a canonical GTPase binding motif (see Figure S1). The first ATG codon (green arrow) after the Alu repeat sequence is used as putative start codon for the open reading frame of *IRGM*. The transcription start site is marked with green flag. FS indicates frameshift mutation. TGA and TAA denote the position of stop codons (arrows). The shaded white, blue and green colors indicate predicted intron, UTR or exon, respectively. The genomic loci are not drawn to scale with the exception of the full-length sequence of *IRGM* ORF.

**Figure 2:**
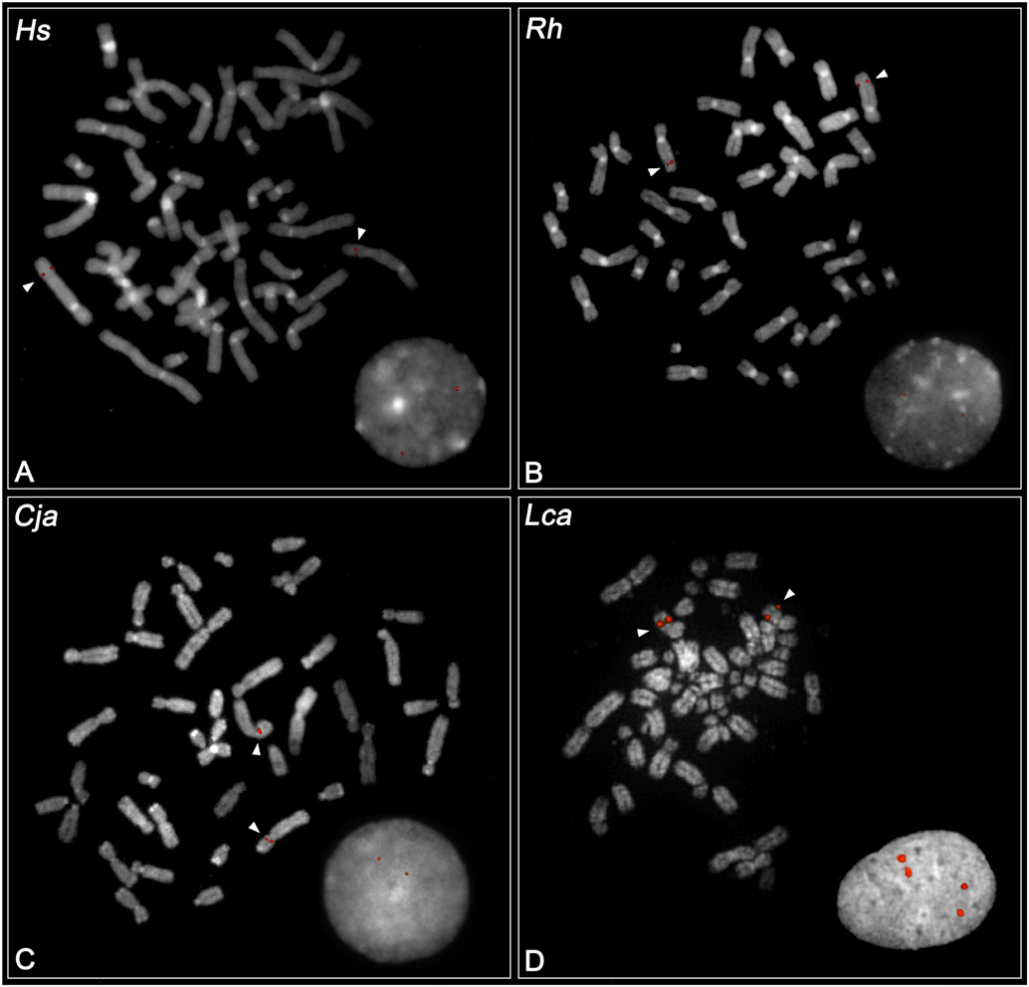
FISH analysis of *IRGM*. The figure shows examples of FISH experiments on Hs (*Homo sapiens*), Rh (*Macaca mulatta*), Cja (*Callithrix jacchus*) and Lca (*Lemur catta*), with the use of human fosmid clone WIBR2-3607H18 (A, B, C) and lemur species-specific BAC clone LB2-77B23 (D).

Sequence analysis of two different prosimian species (*Microcebus murinus* and *Lemur catta*) confirmed the mammalian archetypical organization with three *IRGM* paralogs in each species (Figure 1). FISH analysis showed that genes in these species are organized as part of a tandem gene family similar to the organization observed within the mouse genome (Figure 2). In contrast, FISH and sequence analysis of various monkey and great ape species (see Text S1) confirmed a single copy in each of these species. Based on the estimated divergence of strepsirrhine and platyrrhine primate lineages, we conclude the *IRGM* gene cluster contracted to a single truncated copy 40–50 million years ago within the anthropoid lineage of evolution.

We next compared the structure of the *IRGM* gene in various primate species. One of the three mouse lemur *IRGM* genes (*IRGM9*) preserves a complete ORF based on the mouse model and shows the greatest homology to mouse *Irgm1*. The ORF encodes a putative 47 kD protein including a classical N-terminal region as well as classical motifs at the end of the carboxyl-terminus associated with most functional murine *IRGM* loci [8],[9] (see Text S1). The second mouse lemur gene, *IRGM8*, is likely a pseudogene because of a mutation generating a stop codon within the G domain and a frameshift mutation at the C terminus. The third mouse lemur gene, *IRGM7*, is atypical because it has substitutions in the G domain that disrupt the G1 motif that interacts with the nucleotide phosphates and is highly conserved in P-loop GTPases [10] (Figure S1 and Text S1).

In contrast to mouse and prosimian species, all anthropoid primate lineages show the presence of an AluS_c_ repeat immediately after the splicing acceptor that disrupts the ORF of the sole remaining *IRGM* gene (Figure 1 and S2). Sequencing of the *IRGM* locus in four New World monkey species revealed the presence of the same two stop codons disrupting the ORF of *IRGM* in all species. We similarly identified a common frameshift mutation resulting in premature stop codons within the *IRGM* locus in eleven diverse Old World monkey species suggesting that *IRGM* had become pseudogenized before the radiation of these species. Sequencing of the gene in multiple individuals in the same species (five unrelated Rhesus macaque and baboon) suggested that the frameshift mutations were fixed (Figure S3 and Text S1). In total, these data argue that the *IRGM* locus has been nonfunctional since the divergence of the New World and Old World monkey lineages (35–40 million years ago) likely as a result of an Alu repeat integration event that disrupted the ORF of the gene in the anthropoid ancestor (Figure 1).

In contrast to New World and Old World monkeys, sequencing of the *IRGM* locus in humans and African great ape species reveals a restored, albeit truncated, ORF of ∼20 kD in length. This is consistent with an antiserum raised against peptides from the human *IRGM* protein that detected a specific signal at ∼20 kD by Western blot [11]. In contrast to humans and the African great apes, analysis of the orangutan genome assembly predicted a nonfunctional protein (C to T transition at nucleotide position 150 with respect to the start codon resulting in a premature shared stop codon in the ORF (Figure 1 and Text S1). This is the same substitution identified among all Old World monkey genomes suggesting that ancestral ape species carried a pseudogene. We resequenced the *IRGM* gene in twelve different orangutans and five different gibbon species. Six of the twelve individuals from orangutan and one of the five species from gibbon are heterozygous for the C to T substitution. In addition, we noted that all ape *IRGM* copies also shared a new translation initiation codon with a preferred Kozak sequence immediately after the Alu integration. These data indicate that the gene can exist as either a pseudogene or as a complete 20 kD ORF among these Asian ape lineages as a result of either balancing selection or recurrent mutational events. It will be necessary to examine a larger number of individuals within each species to establish the evolutionary history of this locus among the Asian apes.

We noticed an important structural difference in the gene organization for species that regained putative *IRGM* function when compared to those primates with a pseudogenized version. In the common ancestor of humans and great apes, an ERV9 retroviral element integrated within the 5′ end of the *IRGM* gene (Figure 1). We reasoned that this structural difference may have conferred expression differences and analyzed the RT-PCR expression profile of *IRGM* in human, macaque and marmoset. Full-length cDNA sequencing and 5′ RACE revealed that the human transcription start signal mapped specifically within the ERV9 repeat element (Figure 1 and Figure S4) resulting in the addition of a novel 5′UTR exon and an alternative splice form. Although there are five distinct, alternative splice forms of human *IRGM*, all human copies share this first intron.

**Figure 3:**
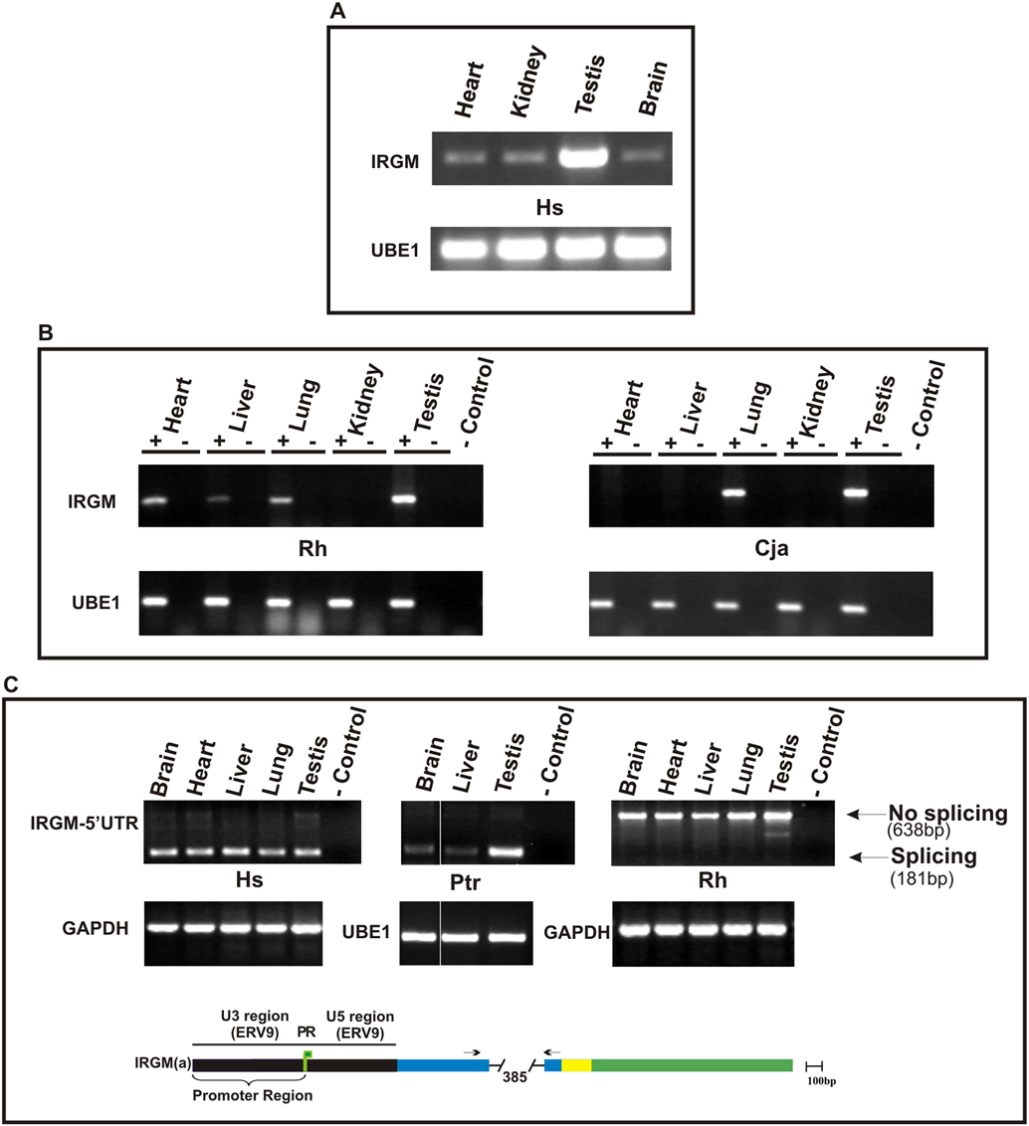
Expression analysis of *IRGM* in human, macaque and marmoset. A) RT-PCR results from cDNA prepared from total RNA extracted from various human (Hs) tissues are compared against [8]. B) rhesus macaque (Rh) and marmoset (*Cja*) tissues. Total RNA was treated with DNAse I prior to cDNA synthesis. cDNA reactions established with (+) and without (−) reverse transcriptase as a control and RT-PCR compared against a positive UBE1 expression control. C) RT-PCR reveals splicing of the 5′ UTR region for human and chimp *IRGM* but not in rhesus macaque. Diagram is showing the promoter and 5′UTR region of *IRGM*. ORF, ERV9, intronic sequence, Alu sequence, and 5′ untranslated region (UTR) depicted in green, black, white, yellow and blue colors respectively. The first ATG codon after Alu sequence is used as putative start codon for the open reading frame of *IRGM*. Transcription start site is marked with green flag. Arrows indicate the position of the primers.

In humans, we observe constitutive levels of expression of *IRGM* in all tissues examined, with the highest expression of *IRGM* in the testis (Figure 3A) [8]. Although *IRGM* does not encode a functional protein in marmoset and macaque, we find evidence of low levels of expression, albeit in a more restricted manner (Figure 3B). Macaque and marmoset, for example, show no expression in the kidney with marmoset *IRGM* expression restricted to testis and lung. Furthermore, we find no evidence in macaque of splicing of the first intron based on the human *IRGM* gene model (Figure 3C) but rather evidence that the first intron remains as a continuous unspliced transcript. We also failed to confirm 3′ downstream splicing events of macaque *IRGM* suggesting that even if stop codons were reverted, a full-length cDNA (comparable to human) could no longer be produced. These data strongly suggest that ERV9 integration significantly reshaped the expression and splicing pattern of *IRGM* in the common ancestor of humans and apes (Figure 3). We note that structural changes of the human *IRGM* locus continues to occur within the human lineage with a 20.1 kb LTR-rich deletion polymorphism, recently identified and sequenced, located 2.82 kb upstream of the ERV9 promoter region [12]. Our preliminary data suggest that this deletion polymorphism alters the relative proportion of alternative splicing of *IRGM* transcripts (Figures S5 and S6).

**Figure 4:**
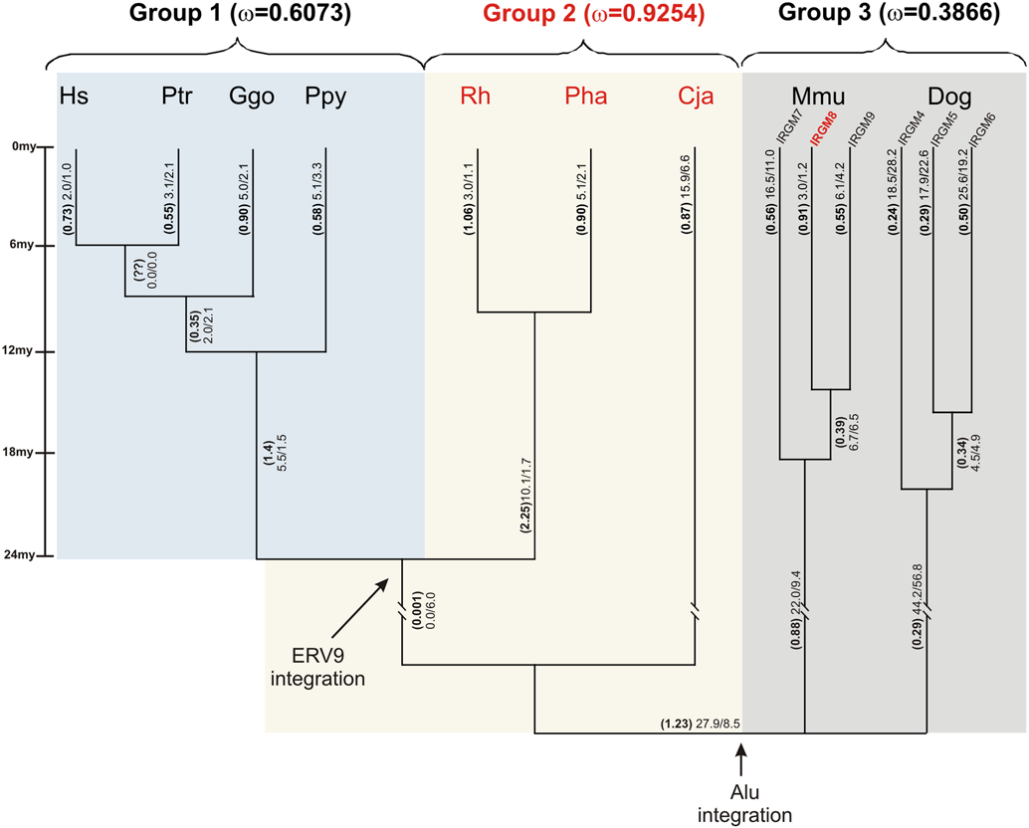
Selection of the IRGM locus during primate evolution. Branch estimates for ω = d_N_/d_S_ are shown using the free branch model in PAML. Species names are indicated as Hs (*Homo sapiens*), Ptr (Chimp, *Pan trogylodytes*), Ggo (*Gorilla gorilla*), Ppy (Orangutan, *Pongo pygmaeus*), Rh (Rhesus macaque, *Macaca mulatta*), Pha (*Papio hamadryas*), Cja (Marmoset, *Callithrix jacchus*), Mmu (*Microcebes murinus*), and Dog (*Canis familiaris*). Omega values (ω) and the number of nonsynonymous and synonymous substitutions (N*d_N_/S*d_S_) for each respective branch are indicated in parentheses. Branch estimates for d_N_/d_S_ are shown for the three groups (see Text S1). Species names highlighted in red carry pseudogenes based on multiple stop codons in the ORF.

We tested for natural selection on *IRGM* coding sequence using maximum likelihood models to estimate evolutionary rates for individual branches in the phylogeny as well as specific codon changes [13],[14]. Based on the structural differences in *IRGM* organization, we first divided our species into three groups: Group 1 consists of species that carry a single copy of *IRGM* with the ERV9 element (human (Hs), chimpanzee (Ptr), gorilla (Ggo) and orangutan (Ppy)); Group 2 consists of species that carry a single copy of *IRGM* but lack the ERV9 element (Macaque (Rh), baboon (Pha) and marmoset (Cja)); while Group 3 was formed by species (dog and mouse lemur) that had multiple copies in a tandem orientation (Figure 4). Phylogenetic branch estimates of d_N_/d_S_ revealed striking differences between Group 2 (ω = 0.9254) and Group 3 (ω = 0.3866) with an intermediate value for Group 1 (ω = 0.6073). Group 3 was found to be under constrained evolution (ω = 0.3866) and it was significantly different (P = 6.09E^−12^) from a model of neutral evolution. In contrast, Group 1 and 2 gene evolutions were indistinguishable from a model of neutral evolution (see Text S1).

## Discussion

**Figure 5:**
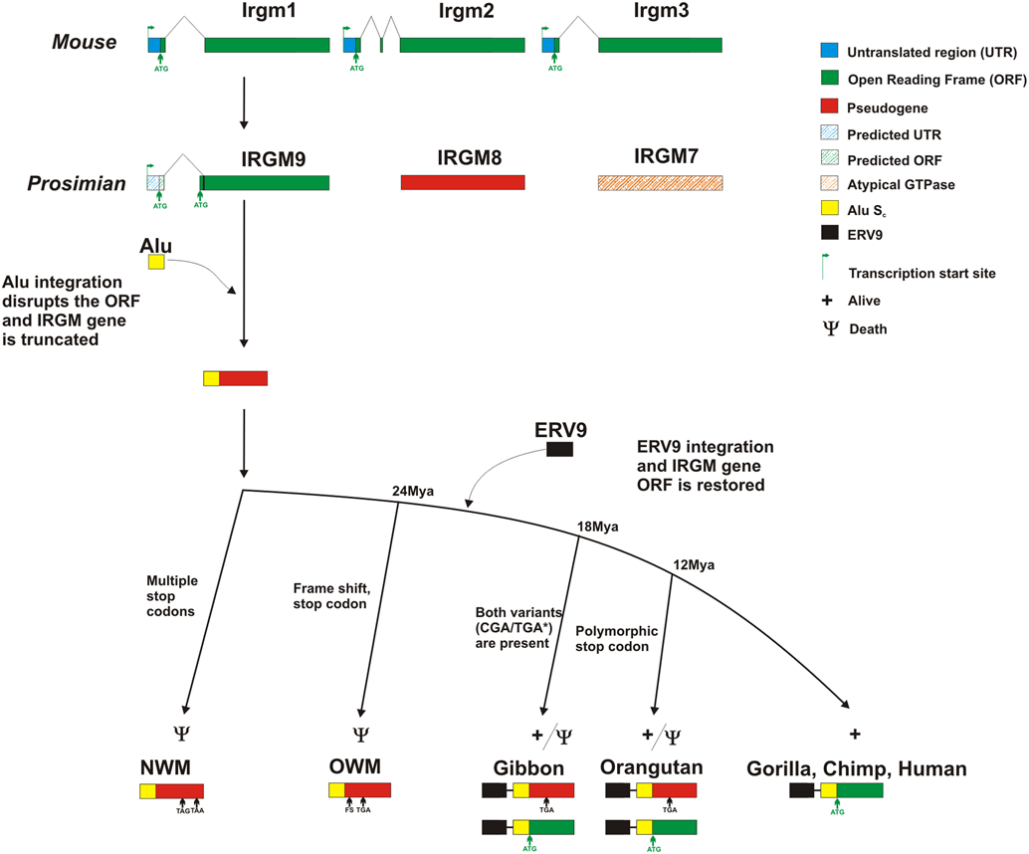
Evolution of *IRGM* loci. A model for the evolution of primate *IRGM* genes is depicted. The mammalian *IRGM* tandem gene family contracts to a single-copy gene after the divergence of prosimians and anthropoids. The single-copy gene is pseudogenized in the anthropoid ancestor due to an AluS_c_ repeat integration into the second exon, disrupting the ORF of the sole remaining *IRGM* gene. Multiple stop codons and frameshift mutations accrue in all Old World and New World monkey lineages. Three mutation events restore the *IRGM* gene in the common ancestor of apes and humans: integration of the ERV9 element to serve as a new promoter, a single-nucleotide mutation that introduces a new ATG codon (green arrow) after the Alu repeat and the loss of a stop codon that is shared with Old World monkey species. The latter event is polymorphic in orangutans rendering both functional and nonfunctional copies in this species. (*) of the five gibbon species analyzed, *H. gabriellae* shows a heterozygote stop codon. In the human and African great ape, the functional copy becomes fixed. Frameshift mutation (Fs) and stop codons are indicated. The genomic loci are not drawn to scale with the exception of the full-length sequence of *IRGM* ORF.

There are two possible interpretations of our results. First, the *IRGM* gene is not functional in humans having lost its role in intracellular parasite resistance ∼40 million years ago when the gene family experienced a contraction from a set of three tandem genes to a sole, unique member whose ORF was disrupted by an AluS_c_ repeat in the anthropoid primate ancestor. In light of the detailed functional studies [11] and the recent associations of this gene with Crohn's disease [6],[7], we feel that this interpretation is unlikely. For example, McCarroll and colleagues recently demonstrated that a 20.1 kb deletion upstream of *IRGM* associates with Crohn's disease as well as the most strongly associated SNP and that the deletion haplotype showed a distinct pattern of *IRGM* gene expression consistent with its putative role in autophagy and Crohn's disease. An alternate scenario is that the *IRGM* gene became nonfunctional ∼40 million years ago (leading to pseudogene copies in Old World and New World monkeys) but was resurrected ∼20 million years ago in the common ancestor humans and apes (Figure 5). In addition to the genetic and functional data, several lines of evidence support this seemingly unusual scenario. First, we find evidence of a restored ORF in humans and African great apes. Second, this change coincided with the integration of the ERV9 element that serves as the functional promoter for the human *IRGM* gene. Such retroposon-induced alterations of gene expression are not without precedent in mammalian species [15],[16]. Third, we find that ape/human codon evolution is consistent with a model of nucleotide constraint resulting in depressed d_N_/d_S_ ratios in the hominid branch (Figure 4) when compared to the Old World and New World species. It is intriguing that the orangutan and gibbon populations possess both a functional and nonfunctional copy of *IRGM*, which would open the possibility to long-term balancing selection or recurrent mutations (see Text S1). The inactivating stop codon is shared with all Old World monkey species suggesting an ancestral event. Moreover, we and others [17] find that the structure of the locus is continuing to evolve in humans altering the expression profiles of *IRGM* transcripts in different tissues. These structural changes are thought to underlie the strong association with Crohn's disease, perhaps, by modulating the efficiency of the autophagic response [17]. Our data suggest remarkable functional plasticity where alleles experience diverse evolutionary pressures over time. Such dynamism in structure and evolution may be critical for a gene family locked in an arms race with an ever-changing repertoire of intracellular parasites.

## Methods

### Sequence Analyses

We retrieved whole genome shotgun sequence of the *IRGM* locus for chimpanzee (*Pan troglodytes*), gorilla (*Gorilla Gorilla*), orangutan (*Pongo pygmaeus*), rhesus macaque (*Macaca mulatta*), marmoset (*Callithrix jacchus*), baboon (*Papio hamadryas*), and Gray Mouse Lemur (*Microcebus murinus*) from NCBI Trace Archive (http://www.ncbi.nlm.nih.gov/Traces/trace.cgi?) and constructed local sequence assemblies using PHRAP (http://www.phrap.org). We sequenced and confirmed the *IRGM* genome organization based on DNA samples from four different New World monkey species and from eleven different Old World monkey species. We also resequenced the *IRGM* gene in unrelated macaques (n = 5), baboons (n = 5), orangutans (n = 12) and gibbons (n = 7). For *Microcebus murinus* with multiple copies of *IRGM*, we first isolated large-insert BAC clones, subcloned and sequenced PCR amplicons corresponding to the different copies.

### FISH Experiments

Metaphase spreads were obtained from lymphoblast or fibroblast cell lines from human (*Homo sapiens*), rhesus macaque (*Macaca mulatta*), marmoset (*Calithrix jacchus*) and lemur (*Lemur catta*). FISH was performed using either human *IRGM* probe WIBR2-3607H18 or lemur *IRGM* BAC DNA LB2-61D22, LB2-77B23 and LB-61A22, directly labeled by nick translation with Cy3-dUTP (Perkin-Elmer). Lemur BAC probes were obtained by library hybridization screening of a *L. catta* genomic library (CHORI Resources: LBNL-2 Lemur BAC Library [http://gsd.jgi-psf.org/cheng/LB2]).

### Expression Analyses

Full-length human *IRGM* transcript was obtained by 5′RACE PCR followed by subcloning (PGEM-T easy) and sequencing (EU742619). RT-PCR experiments were performed using cDNA synthesized (Advantage RT-PCR, Clontech) from mRNA extracted (Oligotex isolation kit, Qiagen) from total RNA (RNA Easy, Qiagen). Total RNA was obtained from tissues isolated from chimpanzee, rhesus macaque, marmoset and human. *IRGM* splice variants were detected by a quantitative PCR assay using the LightCycler SYBR Green System (Roche) with primers *IRGM* (b)-(c)-(d) and *IRGM* all primers (Text S1). Transcript levels were normalized to the amount of the GAPDH and UBE1 transcript, which also served as positive controls for RT-PCR experiments.

### Phylogenetic Analyses

We generated multiple sequence alignments using Clustal-W[18],[19] and constructed neighbor-joining phylogenetic trees (MEGA 3.1) [20]. Tests of selection (ω = d_N_/d_S_) were performed by maximum likelihood using PAML [13] applying the Sites Model [14] to calculate the percentage of codons under positive, neutral evolution or purifying selection and the Branch model [21] to estimate evolutionary pressures at different times during evolution. The Likelihood Ratio Test (LRT) was used to assess the significance of different values of ω for different groups.

## Supporting Information

Figure S1 Amino acid alignment of the *IRGM* proteins. Protein sequence alignment of primate, dog and mouse *IRGM* shows close homology in N-terminal GTPase binding domain (G domain). Canonical GTPase motifs are indicated by red boxes. The sequences are edited to maintain the open reading frame of Cja, Rh, and Pph *IRGM*, which are considered to be pseudogenes (names are indicated in red color). Species names are indicated as: Hs (*Homo sapiens*), Ptr (*Pan trogylodytes*), Ggo (*Gorilla gorilla*), Ppy (*Pongo pygmaeus*), Rh (Rhesus macaque-*Macaca mulatta*), Cja (*Callithrix jacchus*), Pph (*Papio hamadryas*), *IRGM7*, *IRGM8*, *IRGM9* Mmu (*Microcebus murinus*), *IRGM4*, *IRGM5*, *IRGM6* (Dog *IRGM* GMS type GTPases), *IRGM1*, *IRGM2*, *IRGM3* (Mouse *IRGM* GMS type GTPases).
(0.09 MB PDF)

Figure S2 Alignment of the *IRGM* Alu repeat integration region. Blue highlighted sequence denotes the canonical splicing acceptor (based on murine gene model) with the red underlined sequence indicating the position of polypyrimidine tract. Green highlighted sequences correspond to the *IRGM* ORF. Alu integration site is indicated as red box (292 bp). Translation start site with preferred Kozak consensus sequence for Human *IRGM* is indicated as a green arrow. Stop codons in the ORF are indicated as red triangles.
(0.09 MB PDF)

Figure S3 Phylogeny of *IRGM*. Phylogenetic reconstruction of *IRGM* related genes in different primate, dog and mouse species using the NJ method. Species names are indicated as: Mouse (*Mus musculus domesticus*), Dog (*Canis familiaris*), Gray mouse lemur (*Microcebus murinus*), Sbo (*Saimiri boliviensis*), Cge Marmoset (*Callithrix geofroyi*), Cmo (*Callicebus moloch*), Ppi (*Pithecia pithecia*), Mar (*Macaca arctoides*), Mni (*Macaca nigra*), Mmu Rhesus macaque (*Macaca mulatta*), Mfa (*Macaca fascicularis*), Pan (*Papio hamadryas anubis*), Pha Baboon (*Papio hamadryas*), Cce (*Cercopithecus cephus*), Cae (*Cercopithecus aethiops*), Pcr (*Presbytis cristata*), Cpo (*Colobus polykomos*), Cgu (*Colobus guereza*), Hga Gibbon (*Hylobates gabriellae*), Ppy Orangutan (*Pongo pygmaeus*), Ggo Gorilla (*Gorilla gorilla*), Ptr Chimpanzee (*Pan troglodytes*) and Hs Human (*Homo sapiens*). Shared stop codons for New World and Old World monkeys are highlighted in purple and blue respectively. Pseudogenes are highlighted in red.
(0.47 MB PDF)

Figure S4 Alignment of the *IRGM* ERV9 region in (human, chimp, orangutan, macaque and marmoset). Red highlighted sequence denotes the ERV9 element. Yellow and green highlighted sequences correspond to the AluSc element and the *IRGM* ORF. Intron sequence is not included in this alignment indicated as red box (489 bp). Transcription start site (+1) indicated as green box. Stop codons in open reading frame are indicated as red triangles. Note the presence of a marmoset insertion sequence: (TAATGATAATTTCTAATCACTGCAAGAATCACATCACCTTCTTTGAATCAATCTCAAATACCTGGCCTGGTGGGAGCCAGGTTCTGCTCTTCTTCAAGG).
(0.11 MB PDF)

Figure S5 Structural variation and *IRGM* mRNA expression levels. A) A schematic summarizing the location of a sequenced structural polymorphism with respect to the *IRGM* gene (see Figure S6). B) Relative fold expression of *IRGM* mRNA and proportion of splice variants were detected by real-time PCR. Expression data were first normalized against housekeeping gene UBE1 and then cross-compared using the heterozygote as the reference (GM15510 (I/D)). The figure shows the relative fold expression of GM18507 (I/I), GM18555 (D/D) and GM15510 (I/D). C) Relative fold expression of *IRGM* (B) detected by real-time PCR. The figure shows a two-fold expression difference between a lymphoblastoid cell line homozygous for the 20.1 kb insertion GM18507 (I/I) and cell line homozygous for the deletion GM18555 (D/D).
(1.38 MB PDF)

Figure S6 Structural polymorphism 5′ upstream of the *IRGM* locus. A) A miropeats alignment comparing the human chromosome 5 reference sequence to a sequence from an alternate haplotype (AC207974 from HapMap individual NA18956). The alignment depicts a 20.1 kb deletion region 5′ upstream of the human *IRGM*. Arrow indicates the transcription start point within the ERV9 retroviral element. Green box represents *IRGM* open reading frame; red boxes indicate exons for adjacent MST150 gene. B) Array comparative genomic hybridization (aCGH) results for nine human DNA samples (four African and four non-African) against a reference genome DNA sample (NA15510). The analysis confirms a 20.1 kb deletion polymorphism (indicated as red dotted line) located at a distance of 2.82 kb 5′ to the *IRGM* transcription start site. The individual NA15510 is hemizygous (one copy) and is used as the reference in these experiments.
(0.42 MB PDF)

Text S1 Supplementary note: Death and resurrection of the human *IRGM* gene.
(0.61 MB PDF)
