# Supplementary material for: Death and Resurrection of the Human IRGM Gene
Source: PLoS Genet. 2009 Mar 6;5(3):e1000403. doi: 10.1371/journal.pgen.1000403 (PMC2644816; doi:10.1371/journal.pgen.1000403)
Supplement: Figure S1 — Amino acid alignment of the IRGM proteins. Protein sequence alignment of primate, dog and mouse IRGM shows close homology in N-terminal GTPase binding domain (G domain). Canonical GTPase motifs are indicated by red boxes. The sequences are edited to maintain the open reading frame of Cja, Rh, and Pph IRGM, which are considered to be pseudogenes (names are indicated in red color). Species names are indicated as: Hs (Homo sapiens), Ptr (Pan trogylodytes), Ggo (Gorilla gorilla), Ppy (Pongo pygmaeus), Rh (Rhesus macaque-Macaca mulatta), Cja (Callithrix jacchus), Pph (Papio hamadryas), IRGM7, IRGM8, IRGM9 Mmu (Microcebus murinus), IRGM4, IRGM5, IRGM6 (Dog IRGM GMS type GTPases), IRGM1, IRGM2, IRGM3 (Mouse IRGM GMS type GTPases). (0.09 MB PDF) [file pgen.1000403.s001.pdf]

Figure S1. Amino acid alignment of the IRGM proteins

```
Hs_IRGM      1 MNVEKASADGNLPEVISNIKETLKIVSRTPVNITMAGDSGNGMSTFISALRNTGHEGKAS
Ptr_IRGM     1 MNVEKASADGNLPEVISNIKETLKIVSRTPVNITTAGHSGNGMSTFISALRNTGHEGKAS
Ggo_IRGM     1 MNVEKASADENLPEVISNIKETLKIVSRTPVNITMAGDSGNGMSTFISALRNTGHEGKAS
PPY_IRGM     1 MSVEKASADGNLPEVISDIKETLKIVSRTPVNITMAGDSGNGMSTFISALRNTGHEGKAS
Cja_IRGM     1 MNVERASADGDLPEVVSAIKESLKIVFRTPVNIAMAGDSGNSISTFISALQIAGHEAKAS
Rh_IRGM      1 MNVEKALVGGNLPEVASAINETLKIVSRTPVNIAMAGDSGSGMNTFISALRNTGHEGKPS
Pph_IRGM     1 MNGDKALISGNLPYEASAIKETLKIVSSTFFCIAMAENSGSGMNTFIGALRNTGHEGKPS
IRGM9        1 MSIEKAIAGGNLPELVSAVRETVKMVSRTPVNVAVTGDSGNGMSSFINALRNIGHEEEAS
IRGM8        1 MSIEKAIAGGNLPELVSAIRETVKMVSRTPVNVAVTGDSGNGMSSFINALQNIGHEEEAS
IRGM7        1 MSIEKAIAGGNLPEVVSAIRESLKMASRTPVNVALTGNSGNDMSSFINPLRNIGHEEEAS
IRGM5        1 TNIEKALGDGKLLEVVSMIRETLETVSSAPVSIAVTGDSGNGMSSFINALREIGHDEKDS
IRGM6        1 INIEKALGDGKLLEVVSMIRETLETVSSAPVSIAVTGDSGNGMSSFINALREIGHDEKDS
IRGM4        1 MNIEKALGEGKLLDMVSVVRETLETASSVPVSIAVTGDSGNGMSTFINALRKIGHNEEDS
Irgm2        1 KRIETAVKEGEVVKVVSIVKEIIQNVSRNKIKIAVTGDSGNGMSSFINALRLIGHEEKDS
Irgm3        1 EDIGKAVTEGNLQKVIGIVKDEIQSKSRYRVKIAVTGDSGNGMSSFINALRFIGHEEEDS
Irgm1        1 RSTERALREGKLLELVYGIKETVATLSQIPVSIFVTGDSGNGMSSFINALRVIGHDEDAS
```

GXXXXGMS
G1

```
Hs_IRGM     61 PPTELVKATQRCASYFS-SHFSNVVLWDLPGTGSATTTLENYLMEMQFNRYD-FIMVASA
Ptr_IRGM    61 PPTGLVKATQRCASYFS-SHFSNVVLWDLPGTGSATKTLENYLMEMQFNRYD-FIMVASA
Ggo_IRGM    61 PPTGLVKATQKCASYFS-SHFANVVLWDLPGTGFATKTLENYLMEMQFNRYD-FIMVASA
PPY_IRGM    61 PRTGLVKTTQRCASYFS-SHFSNVVLWDLPGTGSATKTLENYLMEMQFNQCD-FIMVASA
Cja_IRGM    61 PPTGLVKATQRCASYFS-SRFPNVVLWDLPGAGSATKTLENYLMEMQFNQYD-FIMVASA
Rh_IRGM     61 PPTGLVKATQRCAPYLS-SHFPNMVLWDLPGTGSATKTLENYLMEMQFNQYD-FIMVASA
Pph_IRGM    61 PPTGLVTPTQKCAPYFS-SPFPHVPRWDLPGIWSANYCLCNYHMEMRVHQYD-FILGASA
IRGM9       61 APVGVLKTTQTHACYLS-PHFPNVVLWDLPGIVSAAQSLENYATEMQFSRYDFFIIIASE
IRGM8       61 APVGVLKTTQTRACYLS-PHFPNVELWDLPGTECAAQSLENYATEMQFSRYDFFIIIASE
IRGM7       61 VPVAVLKTTQTRACYLS-PHFPNVVLWDLPGTECAAQSLENYSMEMQFSRYDFFIIIVSE
IRGM5       61 APTGVVRTTQVPTCYSS-SHFPYMELWDLPGTGTGTQSLENYLEKIHFSQYDLFIIIASE
IRGM6       61 APTGVVRTTQVPTCYSS-SHFPYMELWDLPGTGTGTQSLENYLEKIHFSQYDLFIIIASE
IRGM4       61 APTGVVRTTQIPTCYSF-SDIPNVELWDLPGTGAATQNLETYLEEMQFSKYDLFIIIASE
Irgm2       61 APTGVVRTTQKPTCYFS-SHFPYVELWDLPGLGATAQSVESYLEEMQISIYDLIIIVASE
Irgm3       61 APTGVVRTTKKPACYSSDSHFPYVELWDLPGLGATAQSVESYLEEMQISTFDLIIIVASE
Irgm1       61 APTGVVRTTKTRTEYSS-SHFPNVVLWDLPGLGATAQTVEDYVEEMKFSTCDLFIIIASE
```

DXXG
G3

```
Hs_IRGM    119 QFSMNHVMLAKTAEDMGKKFYIVWTKLDMDLSTGALPEVQLLQ-IRENVLENL
Ptr_IRGM   119 QFSMNHVMLAKTAEDMGKKFYIVWTKLDLDLSTGALPEVQLLQ-IRENVLENL
Ggo_IRGM   119 QFSMNHVMLAKTPEDMGKKFYIVWTKLDMDLSTGALPEVQLLQ-IRENVLENL
PPY_IRGM   119 QFSMNQVMLAKTAEDMGKKFYIVWTKLDMDLSTGALPEVQLLQ-IRENVLENL
Cja_IRGM   119 QFSMNHVILAKTIEDMGKKFYIVWTKLDMDLSTGALPEVQLLQ-IRENVLESL
Rh_IRGM    119 QFSMNHVMLAKTTEDMGKKFYIVWTKLDMDLSTGALPEVQLLR-IRENVLENL
Pph_IRGM   119 QFIMIHVMLDVTTDDMGYKFYIVWTSLDMDLSTGALPDVQLLH-IRLNVLENL
IRGM9      120 QFSMNHVMLAKTVEDMGKQFYIVWTKLDMDLNTSALPKGQLGKIIRENILENL
IRGM8      120 QFSMNHVMLAKTVEDMGKHFYIVWTKLDMDLNTSALPKGQLRQIIRENILENL
IRGM7      120 QFSMNHVILAKTMWDMRKHFYVVWTKLDMDLNTSALPEGQLLQIIRENILENL
IRGM5      120 QFSMNLVKLVKAIQRQGKRFYIVWTKLDRDLSTRVLPEEQVLQNIWENIQETL
IRGM6      120 QFSMNLVKLVKAIQRQGKRFYIVWTKLDRDLSTRVLPEEQVLQNIWENIQETL
IRGM4      120 QFSMNLVKLVKSIQGQGKRFYIVWTKLDRDLSTCVLSEEQLLRNIRENIRETL
Irgm2      120 QFSLNHVKLAITMQRMRKRFYVVWTKLDRDLSTSTFPEPQLLQSIQRNIRDSL
Irgm3      121 QFSSNHVKLAITMQRMRKRFYVVWTKLDRDLSTSTFPEPQLLQSIQRNIRENL
Irgm1      120 QFSSNHVKLSKIIQSMGKRFYIVWTKLDRDLSTSVLSEVRLLQNIQENIRENL
```

TKXD
G4
