# Supplementary material for: Death and Resurrection of the Human IRGM Gene
Source: PLoS Genet. 2009 Mar 6;5(3):e1000403. doi: 10.1371/journal.pgen.1000403 (PMC2644816; doi:10.1371/journal.pgen.1000403)

Figure S2. Alignment of the *IRGM* Alu repeat integration region

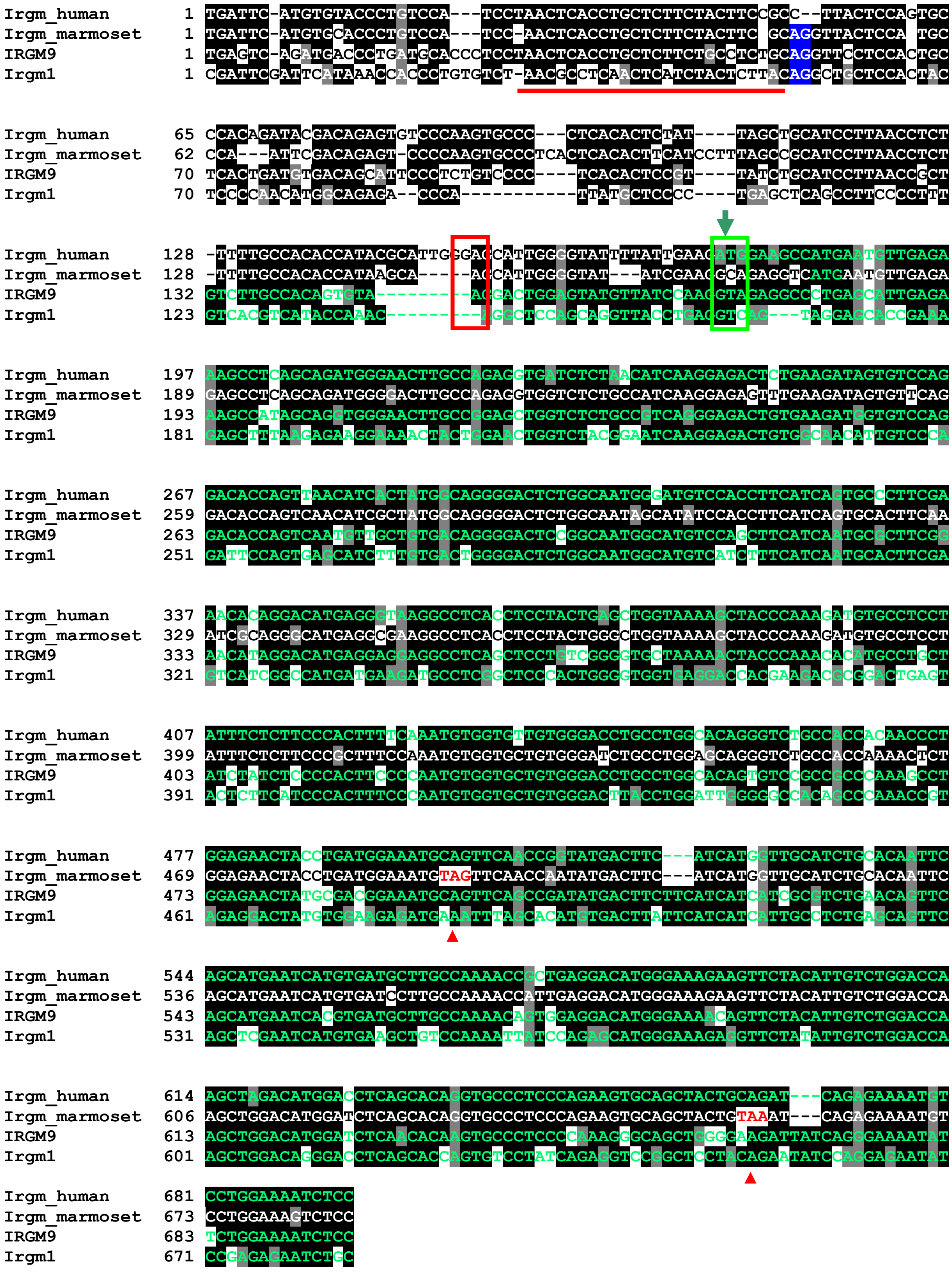

Supplement: Figure S2 — Alignment of the IRGM Alu repeat integration region. Blue highlighted sequence denotes the canonical splicing acceptor (based on murine gene model) with the red underlined sequence indicating the position of polypyrimidine tract. Green highlighted sequences correspond to the IRGM ORF. Alu integration site is indicated as red box (292 bp). Translation start site with preferred Kozak consensus sequence for Human IRGM is indicated as a green arrow. Stop codons in the ORF are indicated as red triangles. (0.09 MB PDF) [file pgen.1000403.s002.pdf]
