# Supplementary figures and images for: Death and Resurrection of the Human IRGM Gene
Source: PLoS Genet. 2009 Mar 6;5(3):e1000403. doi: 10.1371/journal.pgen.1000403 (PMC2644816; doi:10.1371/journal.pgen.1000403)

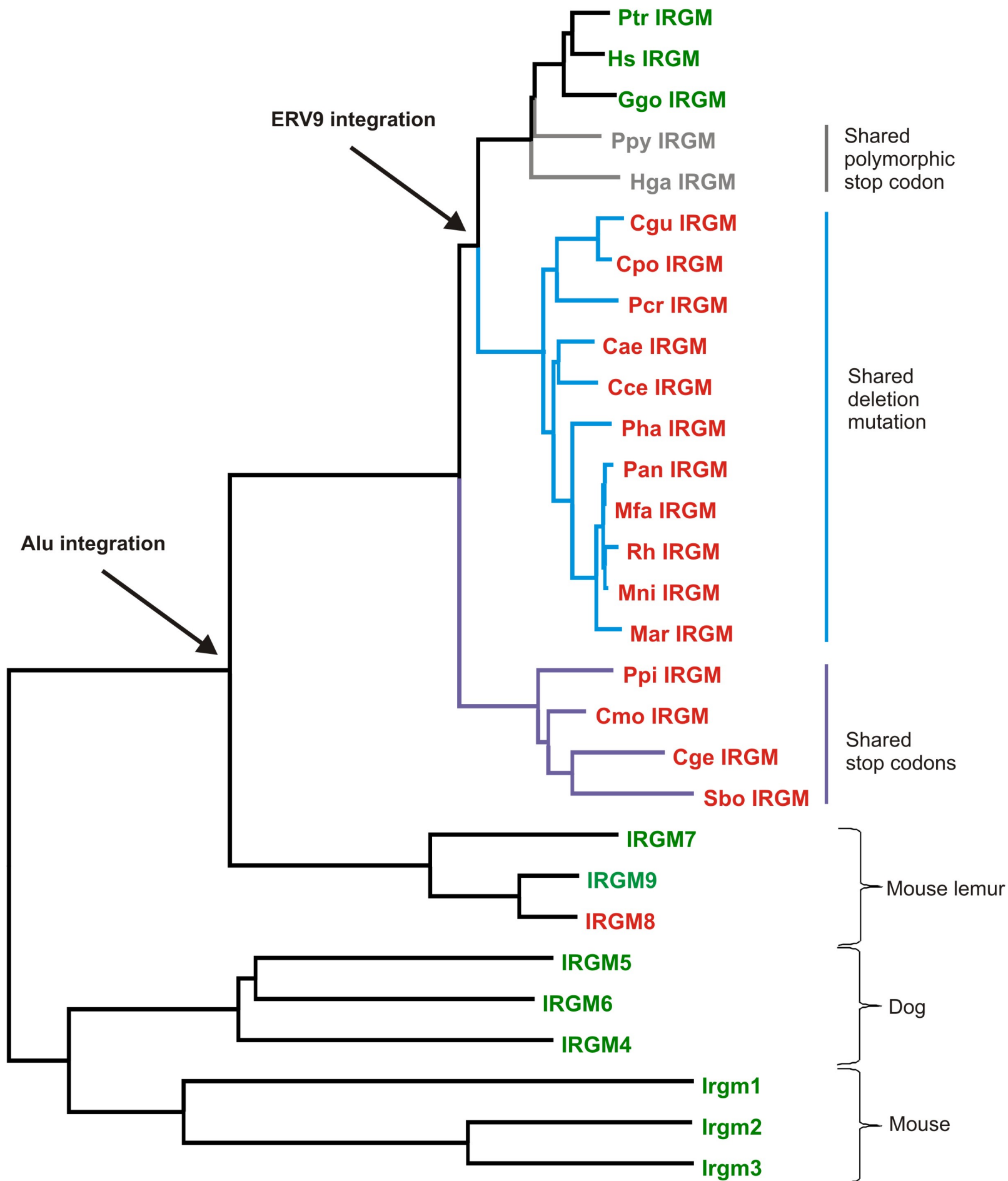

0.02

Supplement: Figure S3 — Phylogeny of IRGM. Phylogenetic reconstruction of IRGM related genes in different primate, dog and mouse species using the NJ method. Species names are indicated as: Mouse (Mus musculus domesticus), Dog (Canis familiaris), Gray mouse lemur (Microcebus murinus), Sbo (Saimiri boliviensis), Cge Marmoset (Callithrix geofroyi), Cmo (Callicebus moloch), Ppi (Pithecia pithecia), Mar (Macaca arctoides), Mni (Macaca nigra), Mmu Rhesus macaque (Macaca mulatta), Mfa (Macaca fascicularis), Pan (Papio hamadryas anubis), Pha Baboon (Papio hamadryas), Cce (Cercopithecus cephus), Cae (Cercopithecus aethiops), Pcr (Presbytis cristata), Cpo (Colobus polykomos), Cgu (Colobus guereza), Hga Gibbon (Hylobates gabriellae), Ppy Orangutan (Pongo pygmaeus), Ggo Gorilla (Gorilla gorilla), Ptr Chimpanzee (Pan troglodytes) and Hs Human (Homo sapiens). Shared stop codons for New World and Old World monkeys are highlighted in purple and blue respectively. Pseudogenes are highlighted in red. (0.47 MB PDF) [file pgen.1000403.s003.pdf]

(a)

Structural variation and Gene structure

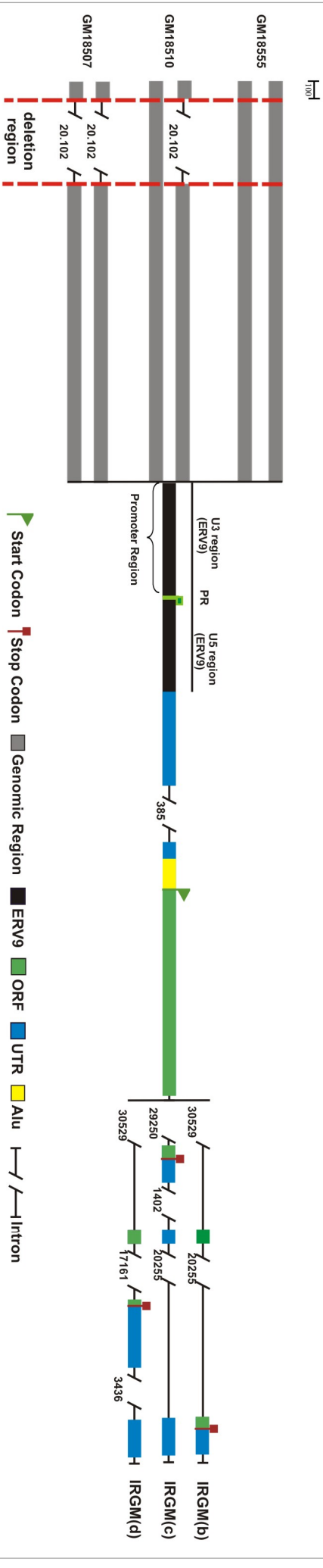

(b)

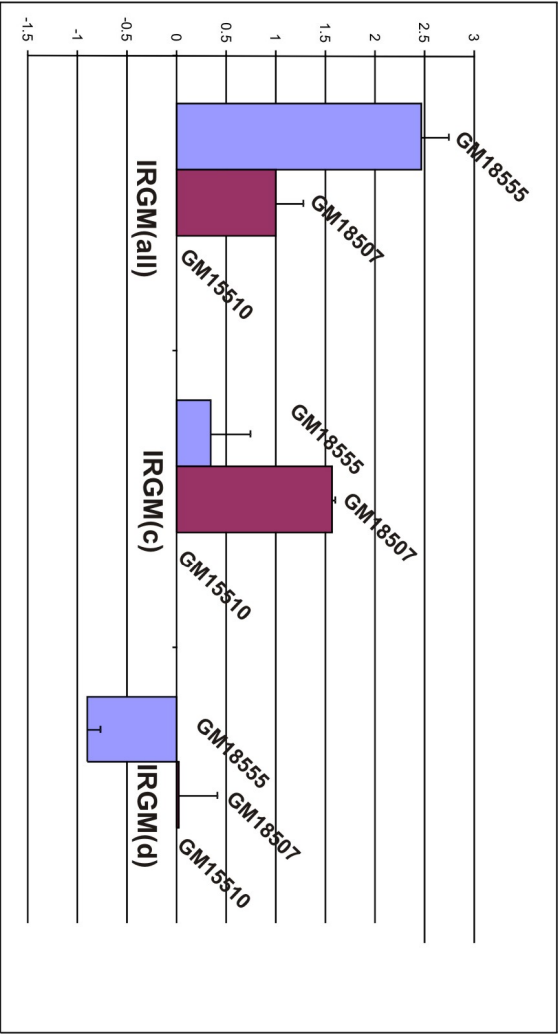

(c)

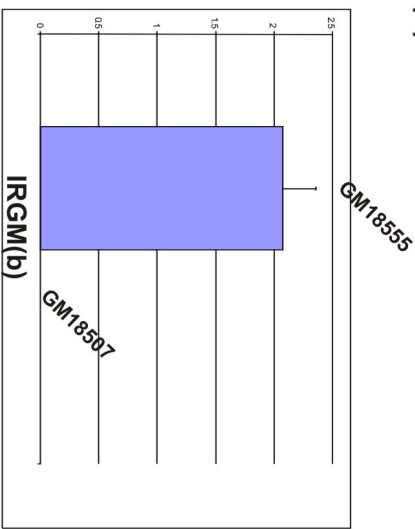

Supplement: Figure S5 — Structural variation and IRGM mRNA expression levels. A) A schematic summarizing the location of a sequenced structural polymorphism with respect to the IRGM gene (see Figure S6). B) Relative fold expression of IRGM mRNA and proportion of splice variants were detected by real-time PCR. Expression data were first normalized against housekeeping gene UBE1 and then cross-compared using the heterozygote as the reference (GM15510 (I/D)). The figure shows the relative fold expression of GM18507 (I/I), GM18555 (D/D) and GM15510 (I/D). C) Relative fold expression of IRGM (B) detected by real-time PCR. The figure shows a two-fold expression difference between a lymphoblastoid cell line homozygous for the 20.1 kb insertion GM18507 (I/I) and cell line homozygous for the deletion GM18555 (D/D). (1.38 MB PDF) [file pgen.1000403.s005.pdf]

(a)

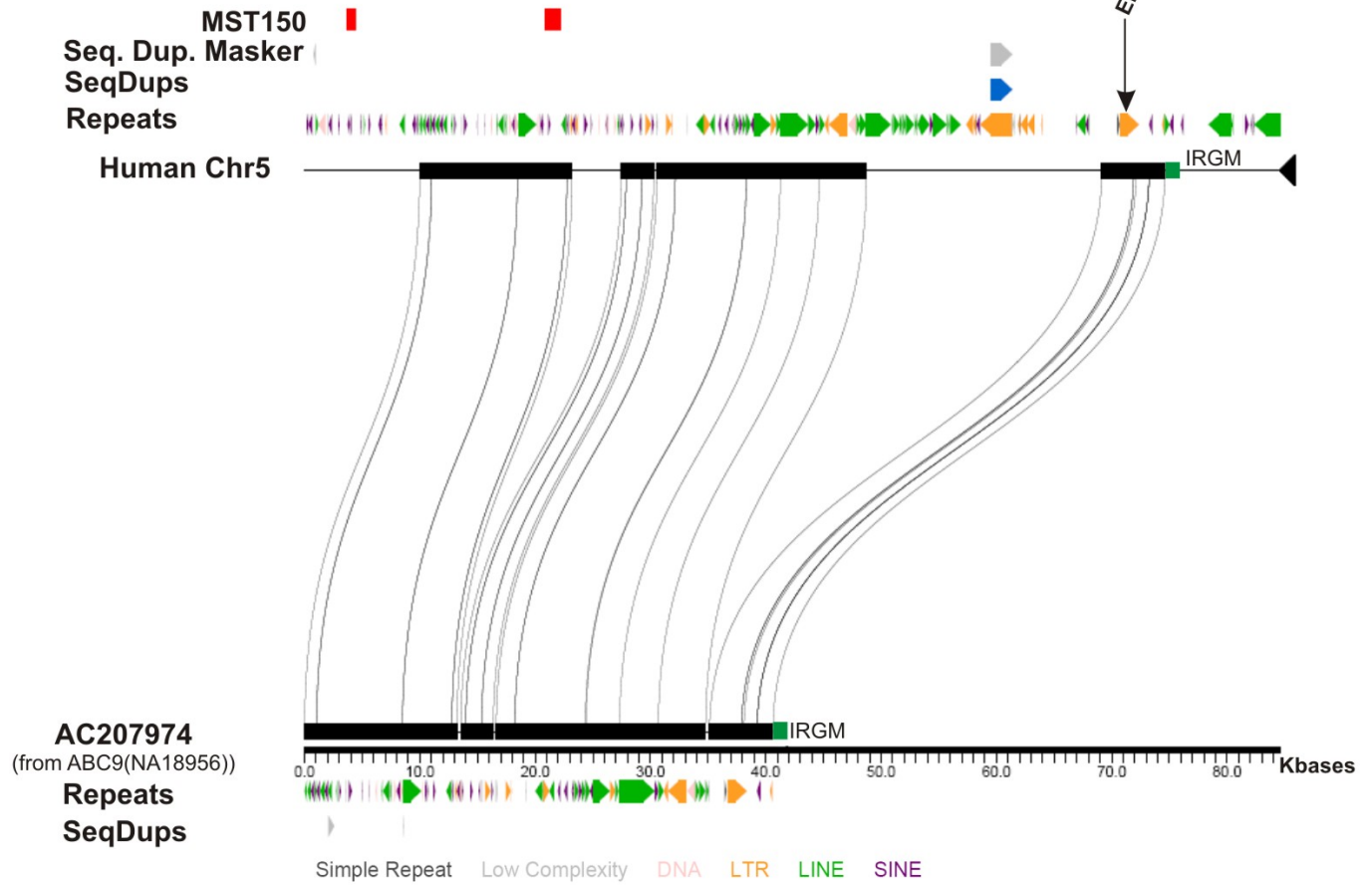

(b)

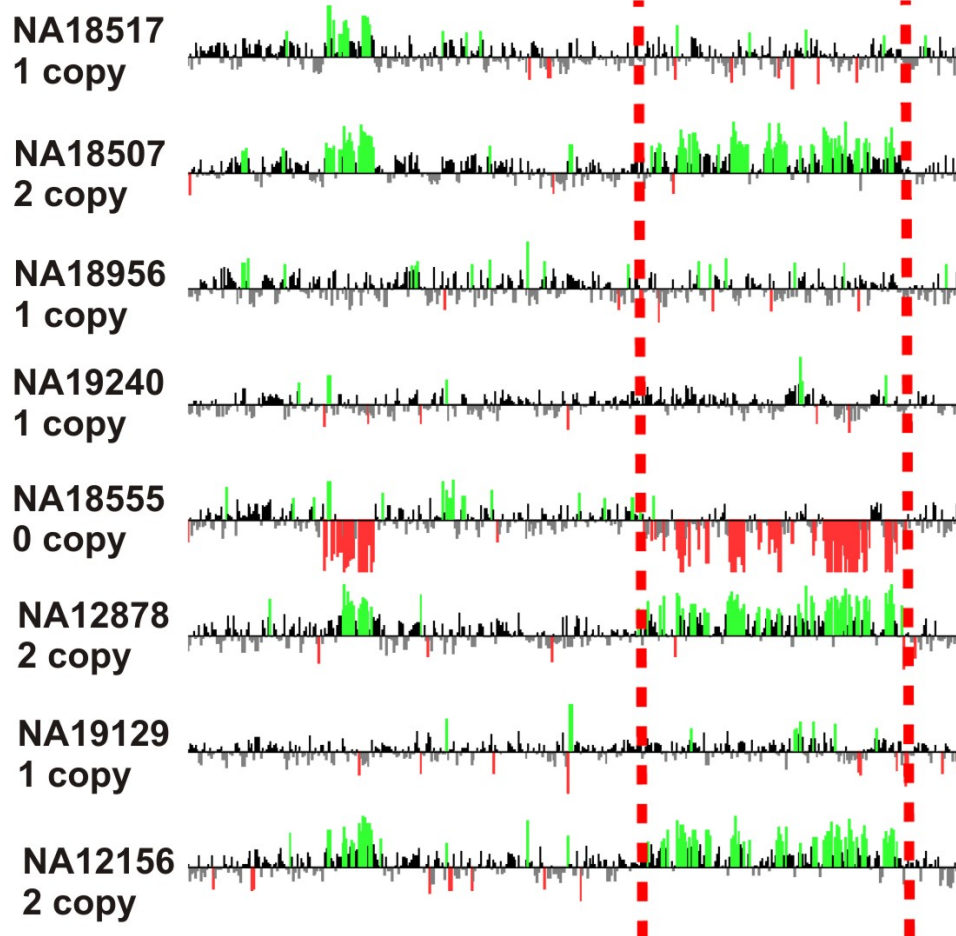

Supplement: Figure S6 — Structural polymorphism 5′ upstream of the IRGM locus. A) A miropeats alignment comparing the human chromosome 5 reference sequence to a sequence from an alternate haplotype (AC207974 from HapMap individual NA18956). The alignment depicts a 20.1 kb deletion region 5′ upstream of the human IRGM. Arrow indicates the transcription start point within the ERV9 retroviral element. Green box represents IRGM open reading frame; red boxes indicate exons for adjacent MST150 gene. B) Array comparative genomic hybridization (aCGH) results for nine human DNA samples (four African and four non-African) against a reference genome DNA sample (NA15510). The analysis confirms a 20.1 kb deletion polymorphism (indicated as red dotted line) located at a distance of 2.82 kb 5′ to the IRGM transcription start site. The individual NA15510 is hemizygous (one copy) and is used as the reference in these experiments. (0.42 MB PDF) [file pgen.1000403.s006.pdf]
