# Supplementary material for: Death and Resurrection of the Human IRGM Gene
Source: PLoS Genet. 2009 Mar 6;5(3):e1000403. doi: 10.1371/journal.pgen.1000403 (PMC2644816; doi:10.1371/journal.pgen.1000403)
Supplement: Figure S4 — Alignment of the IRGM ERV9 region in (human, chimp, orangutan, macaque and marmoset). Red highlighted sequence denotes the ERV9 element. Yellow and green highlighted sequences correspond to the AluSc element and the IRGM ORF. Intron sequence is not included in this alignment indicated as red box (489 bp). Transcription start site (+1) indicated as green box. Stop codons in open reading frame are indicated as red triangles. Note the presence of a marmoset insertion sequence: (TAATGATAATTTCTAATCACTGCAAGAATCACATCACCTTCTTTGAATCAATCTCAAATACCTGGCCTGGTGGGAGCCAGGTTCTGCTCTTCTTCAAGG). (0.11 MB PDF) [file pgen.1000403.s004.pdf]

Figure S4. Alignment of the IRGM including ERV9 region in (human, chimp, orangutan, macaque and marmoset)

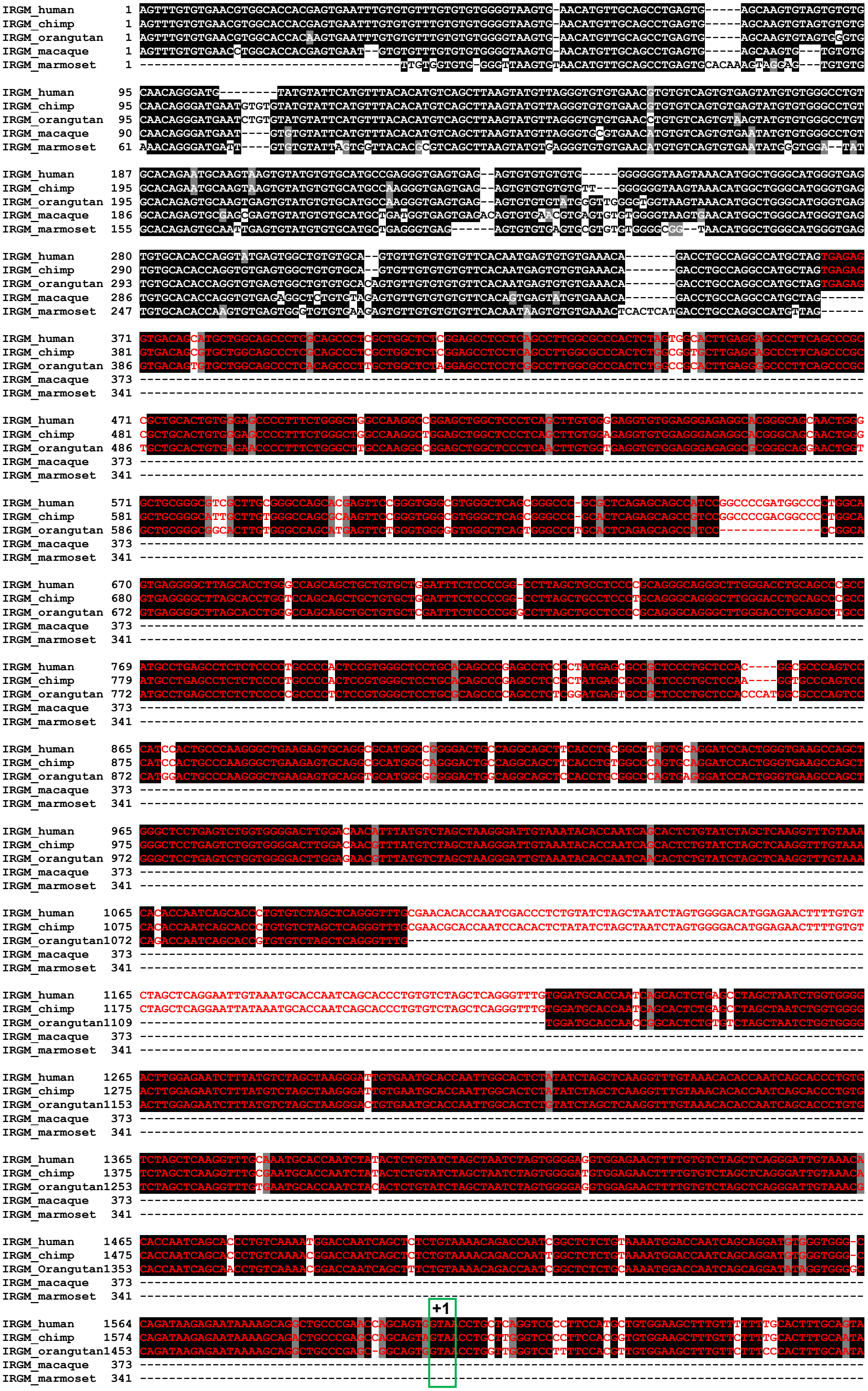

```
IRGM_human     1664 AATCTTGCTGCTGCTCATTCTTTGGGTCCACACTGCCTTTATGAGCTGTAACACTCACCGTGAAGGTCTGCAGCATCACTCCTGAAACCTGTGAAACAAC
IRGM_chimp     1674 AATCTTAG-GCTGCTCATTCTTTGGGTCCACACTGCCTTTATGAGCTGTAACACTCACGGCGAAAGTCTGCAGCATCACTCCTGAAGCCAGTAAAACAAC
IRGM_orangutan1552 AATCTTGCTGCTGCTCAGTCTGTGGGTCCACACTGCTTTAATGAGCTCTAACACTCACTGCAAACGTCTGCAGCTTCACTCCTGAAGCCAGTGAGACCAT
IRGM_macaque   373 ----------------------------------------------------------------------------------------------------
IRGM_marmoset  341 ----------------------------------------------------------------------------------------------------

IRGM_human     1764 GAACCCCCCGGGGGAAACGAACAACTCCAGACTCGCCGCCTGAAGAGCTGTAACTCTCACTTCGAAGGTCTGCAGCTTCGCTCCTGAGTCAGTGAAACC
IRGM_chimp     1773 GAACCCACCGGGAG-AAACGAACAACTCCAGACTCGCCACCTCAAGAGCTGTAACACTCACCTCGAAGGTCTGCAGCTTCACTCCCGAGCCAGTGAAACC
IRGM_orangutan1652 GAACCCACCGGGAG-AAACCAACAACTCCAGACCCGCCACCTTAAGAGCTATAACCGTCACTACAAAGGTCTGCAGCTTTGCTCCTGAGCCAGCGACACC
IRGM_macaque   373 ----------------------------------------------------------------------------------------------------
IRGM_marmoset  341 ----------------------------------------------------------------------------------------------------

IRGM_human     1864 ACGAACCCACCGGAAGGAAGAAACTCTGAACACATCCAAACATCAGAAGGAACAAACTCCGGACACGCAGCCTTTAAGAATTGTAACACTCACCGCGAGG
IRGM_chimp     1872 ACGAACCCACCGGAAGGAAGAAACTCTGAACACATCCAAACGTCAGAAGGAACAAACTCCAGACACGCAGCCTTTAAGAACTGTAACACTCACTGCGAGG
IRGM_orangutan1751 ACGAACCCACGAGAAGGAAGAAACTCAGAACACATCCAAACGTCAGAAGGAACAAACTCCGGACACATGGCCTTTAAGAACTGTAACACTCACCG-----
IRGM_macaque   373 ----------------------------------------------------------------------------------------------------
IRGM_marmoset  341 ----------------------------------------------------------------------------------------------------

IRGM_human     1964 GTCCGTGGCTTCATTCTTGAAGTCAGTGAGACGAAGAACCCACCAATTCCGGTAGGAGTAGGAAATCACATCACCTCCTTTTAATCAGTCTCAAATACCT
IRGM_chimp     1972 GTCCGTGGCTTCCTTCTTGAAGTCAGTGAGAGGAAGAACCCACCAATTCCCGTAGGAGTAGGGAATCACATCACCTTCTTTTAATCAGTCTCAAATACCT
IRGM_orangutan1846 -------GCTTCATTCTTGAAGTCAGTGACACCAAGATCCCACCAGTTCCACTAGGAGTAGAGAATCACATCACCTTCTTTTAATCAGTCTCAA-TACCT
IRGM_macaque   373 -------------------------------------------------------GAGTAGAGAATCACATCACCTTCTTTTAATCAGTCTCAAATACCT
IRGM_marmoset  341 -------------------------------------------------------GTTAGGAGAATTACATCACCTTCTTTTAATCAGTCTCAAATACCT

IRGM_human     2064 GGCCCCCTGTGGAACCTGCAAGAGCCAGGTTTGGCGAATGCAATGATAAGCAATTGTCTAATCACTGCCTTGAAAAAGAGCAGAGCATTTGAGTATGCTG
IRGM_chimp     2072 GGCCTCTTGTGGAACCTGCAAGAGCCAGGTTTGGCGAATGCAATGATAAGCAATTGTCTAATCACTGCCTTGAAAAAGAGCAGAGCATTTGAGTATGCTG
IRGM_orangutan1938 GGCCCT---TGGGAACTGCAAGAGCCAGGTTTGGCGAATGCAATGATAATCAAT-GTCTAATCACTGCCT--GAAAAGAGCAGAGCATTTGAGTATGCTC
IRGM_macaque   418 GGCCCCGTGGGAACCTGCAAGAGCCGGGTTTGGCAA---CAGTGATAATCAATTGTTTAATCACTGCCTCGAAGAAGACCAGAGCATTTGAGTATACTC
IRGM_marmoset  386 GCCTCCATGGAGAACCTGCAAGAGCCAGGTTTGGCGAATGCAGTGATAATCAATTGTGTGATCACTGCCTTGAACAAGAGCAGAGCATTTGAGTATACTC

IRGM_human     2164 TCTCCTCCTCTCCCTCACTTCAGTTGGGCCCAACATGACAGTCTTAATGGAAGCT-CGGGGGTCAAAGGCTGGTGGCTTCCCTCACGTATATTGCAGCAT
IRGM_chimp     2172 TCTCCTCCTCTCCCTCACTTCAGTTGGGCCCAACATGACAGTCTTAATGGAAGCG-CGGGCGTCAAAGGCTGGTGGCTTACTTCATGTATATTGCAGCAT
IRGM_orangutan2032 TCTCGTTCTTTCC-TTACTTCAGTTGGGCCCAACATGACAATCT-AATGGAAGCT-CAGGGGTCAAAGGCTGGTGGCTTACTTCGTATATATTGCAGCAT
IRGM_macaque   515 TCTCCT---CTCCCTCACTTCAGTTGGGCCCAACGTGAGAATCCTAAGGGAAGCTCTGGGGGTCAAAGGCTGGTGGCTTACTTTGTATATATTGCAGCAT
IRGM_marmoset  486 TCTCCTTCTCTCCCCCGCTTCAGTTGGGCCCAATATGACAATCCTAATGGAAGCT-TGGGGGTCAAAGGCAGGTGGCTTACTTCATATATATTGCAGCAT

IRGM_human     2263 TTCAGTACTGC-----TCTGC------ACGTGTGCTCCCCCGCCTGATGAGCTTACTCCAGTGCCCACAGATACGACAGAGTGTCCCAAGTGCCC---CT
IRGM_chimp     2271 TTCGGTACTTC-----TCTGC------ACGTGTGCTCCCCCGACTGATGAGCTTACTCCAGTGCCCACAGATACGACAGAGT-TCCCGAGTGCCC---CT
IRGM_orangutan2129 TTCAGTACTGC-----TCTGC------ACGTGTGCGCCTCTGCCTGATGAGCTTACTTCAGTGCCCACAGATACGACAGAGT-TCCCAAGTGCCC---CT
IRGM_macaque   612 TTCAGTACTGC-----TCTGC------ACGTGTGCTCCC---------CAGCTTACTCCAGTGCCCACCGATACGACAGAGT-TCCCACGTGCCC---CT
IRGM_marmoset  585 TTCAGGACTGCAATCCCTGCCTGATGAGGTAAGTTAAGA-GTTAAAACACGTTACTCCAGTGCCCA---ATTCGACAGAGT-CCCCAAGTGCCCTCACT

IRGM_human     2349 CACACTCTAT----TAGCTGCATCCTTAACCTCTTTTTGCCACACCATACGCATTGGG--------TTTTGTTTGTTTTGAGATGGAGTCTTGCTCTGTT
IRGM_chimp     2356 CACACTCTAT----TAGCTGCATCCTTAACCTCTTTTTGCCGCACCATAAGCATTGGGGTT----TTTTTGTTTGTTTTGAGATGGAGTCTTGGTCTGTT
IRGM_orangutan2214 CACAGTCCAT----TAGCTGCATCCCTAACGTCTTTTTGCCACACCATAAGCATTGGGGTTGGTTTGTTTGTTTGTTTTGAGACGGAGTCTCGCTCTGTT
IRGM_macaque   688 CACACTCCAT----TAGCTGCATCCTAAACC-CTTTTTGCCACACCATAAGCATTGGG-TTTTTTTGTTTGTTTGTTTTGAGACGGAGTCTCGTTCTGGC
IRGM_marmoset  680 CACACTTCATCCTTTAGCCGCATCCTTAACCTCTTTTTGCCACACCATAAGCATT----TTTTTTT---------TCCTGAGATGGAGTTTCACTCTGTC

IRGM_human     2437 GCCAGGCTGGAGTGCAATGGCGTGATCTCAGCTCACTGCAATATCTGCGTCCAGGGTTCAAGCGATTCCCCTGCCTCAGCCTCCTGTATTAGCTGGGACT
IRGM_chimp     2448 GCCAGGCTGGGGTGCAATGGCGTGATCTCAGCTCACTGCAATATCTGCCTCCAGGGTTCAAGCGATTCCCCTGCCTCAGCCTCTTGTATTAGCTGGGACT
IRGM_Orangutan2310 GCCAGGCAGGAGTGCAATGGTGTGACCTCGGCTCACTGCAATATCTGCCTCCTGGGTTCAAGCGATTCCCCTACCTCAGCCTCCTG-ATTAGCTGGGACT
IRGM_macaque   782 T-------GGAGTGCAGTGGCGTGATCCCGGCTCACTGCTACCTCTGCCTCCGAGGTTCGAGTGATTCCCCTGCCTCAGCCTCCTG-ATTAGCTGGGACT
IRGM_marmoset  767 TCT----CAGGCTGGAGTGCAGTGGTTTCAGCTCACTGCAGCTTCCATCTCTTGGGTCCAAGCGAGTCTCCTGCCTCAGCCTCCTG-AGTAGCTGGGACT

IRGM_human     2537 ACAGGCGCACACCACCACGCGCAGCTAATTTTTTTGTATTTTAGTAGAGAA--GGTTTCACGATGTTGGCCAGGATGGTCTCAATCTCCTGACCTC--GT
IRGM_chimp     2548 ACAGACACACACCACCACGCCCAGCTAATTTTTTTGTATTTTAGTAGAGAC--GGTTTCACGATGTTGGCCAGGATGGTCTTGATCTCCTGACCTC--GT
IRGM_orangutan2409 ACAGGCGCACACCACCACGCCCAGCTAATTTTTT-GTATTTTAGTAGAGACAGGGTTTCACCATGTTGGTCAGGATGGTCTCGATCTCCTGACCTC--GT
IRGM_macaque   874 ACAGGCGCACACCACCACACCCGGCTAATTTTTT-GTATTTTAGTAGAGACAGGGTTTCACCATGTTGGCCAGGATGGTCTCAATCTCCTGACCTC--GT
IRGM_marmoset  862 ACAGGCATGCACCACCACACCTGGCTAATTTTTT-GTATTTTAGTAGGGACGGGGTTTCCCCATGTTGGCCAAACTGATCTGGAACTCCTGACCTCAGGT

IRGM_human     2633 GATCTGCTCGCCTCGGCCTTCCAAAGTGCTGGGATTACAGGCATGAGCCACGGCGCTTGGCCAGCATTGGGGTATTTTATTGAAGATGGAAGCCATGAAT
IRGM_chimp     2644 GATCTGCTCGCCTCGGCCTTCCAAAGTGCTGGGATTACAGGCATGAGCCACGGCGCCTGGCCGGCATTGGGGTATTTTCTTGAAGATGGAAGCCATGAAT
IRGM_orangutan2506 GATCCGCTTGCCTTGGCCTTCCAAAGTGCTGGGATTACAGGCATGAGCCATGGTGCCCGGCCAGCATTGGGGTATTTTATTGAGGATGGAGGCCATGACT
IRGM_macaque   971 GGTCCGCTCGCCTCAGCCTCCCAAAGTGCTGGGATTATAGGCAGGAGCCACCGCACCCGGCCAGCATTGGGGTATTTTATCAAAGACGGAGGCCACGAAT
IRGM_marmoset  961 GATCCACCTGCTTTGGCCTCCCAAAATGCCAGGATTACAGGTATGAGCCACCACGCCCAGCCAGCATTGGGGTA---TATCGAAGGCAGAGGTCATGAAT

IRGM_human     2733 GTTGAGAAAGCCTCAGCAGATGGGAACTTGCCAGAGGTGATCTCTAACATCAAGGAGACTCTGAAGATAGTGTCCAGGACACCAGTTAACATCACTATGG
IRGM_chimp     2744 GTTGAGAAAGCCTCAGCAGATGGGAACTTGCCAGAGGTGATCTCTAACATCAAGGAGACTCTGAAGATAGTGTCCAGGACACCGGTTAACATCACTACGG
IRGM_orangutan2606 GTTGAGAAAGCCTCAGCAGATGGGAACTTGCCAGAGGTGATCTCTGACATCAAGGAGACTCTGAAGATAGTGTCCAGGACACCAGTCAACATCACTATGG
IRGM_macaque   1071 GTTGAGAAAGCCTTAGTAGGTGGGAACTTGCCAGAGGTGGCCTCTGCCATCAACGACACTCTGAAGATAGTGTCCAGGACACCAGTCAACATTGCTATGG
IRGM_marmoset 1058 GTTGAGAGAGCCTCAGCAGATGGGGACTTGCCAGAGGTGGTCTCTGCCATCAAGGAGAGTTTGAAGATAGTGTTCAGGACACCAGTCAACATCGCTATGG

IRGM_human     2833 CAGGGGACTCTGGCAATGGGATGTCCACCTTCATCAGTGCCCTTCGAAACACAGGACATGAGGGTAAGGCCTCACCTCCTACTGAGCTGGTAAAAGCTAC
IRGM_chimp     2844 CAGGGCACTCTGGCAATGGGATGTCTACCTTCATCAGTGCCCTTCGAAACACAGGACATGAGGGTAAGGCCTCACCTCCTACTGGGCTGGTAAAAGCTAC
IRGM_orangutan2706 CAGGGGACTCTGGCAATGGGATGTCCACCTTCATCAGTGCCCTTCGAAACACAGGACATGAGGGGAAGGCCTCACCTCGTACTGGGCTGGTAAAAACTAC
IRGM_macaque   1171 CAGGG-ACTCTGGCAGTGGGATGAACACCTTCATCAGTGCCCTTTGAAACACAGGACATGAGGGGAAGCCCTCACCTCCTACTGGGCTGGTAAAAGCTAC
IRGM_marmoset 1158 CAGGGGACTCTGGCAATAGCATATCCACCTTCATCAGTGCACTTCAAATCGCAGGGCATGAGGCGAAGGCCTCACCTCCTACTGGGCTGGTAAAAGCTAC

IRGM_human     2933 CCAAAGATGTGCCTCCTATTTCTCTTCCCACTTTTCAAATGTGGTGTTGTGGGACCTGCCTGGCACAGGGTCTGCCACCACAACCCTGGAGAACTACCTG
IRGM_chimp     2944 CCAAAGATGTGCCTCCTATTTCTCTTCCCACTTTTCAAATGTGGTGTTGTGGGACCTGCCTGGCACAGGGTCTGCCACCAAAACCCTGGAGAACTACCTG
IRGM_orangutan2806 CCAAAGATGTGCCTCCTATTTCTCTTCCCACTTTTCAAATGTGGTGTTGTGGGACCTCCCTGGCACAGGGTCTGCCACGAAAACCCTGGAGAACTACCTG
IRGM_macaque   1270 CCAAAGATGTGCCCCTTATTTATCTTCCCACTTTCCAAATATGGTGCTGTGGGACCTGCCTGGCACAGGGTCTGCCACCAAAACCCTGGAGAACTACCT
IRGM_marmoset 1258 CCAAAGATGTGCCTCCTATTTCTCTTCCCGCTTTCCAAATGTGGTGCTGTGGGATCTGCCTGGAGCAGGGTCTGCCACCAAAACTCTGGAGAACTACCTG

IRGM_human     3033 ATGGAAATGCAGTTCAACCGGTATGACTTCATCATGGTTGCATCTGCACAATTCAGCATGAATCATGTGATGCTTGCCAAAACCGCTGAGGACATGGGAA
IRGM_chimp     3044 ATGGAAATGCAGTTCAACCGGTATGACTTCATCATGGTTGCATCTGCACAATTCAGCATGAATCATGTGATGCTTGCCAAAACCGCTGAGGACATGGGAA
IRGM_orangutan2906 ATGGAAATGCAGTTCAACCAGTGTGACTTCATCATGGTTGCATCTGCACAATTCAGCATGAATCAGGTGATGCTTGCCAAAACCGCTGAGGACATGGGAA
IRGM_macaque   1370 ATGGAAATGCAGTTCAACCAGTATGACTTCATCATGGTTGCATCTGCACAATTCAGCATGAATCATGTGATGCTTGCCAAAACCACTGAGGACATGGGAA
IRGM_marmoset 1358 ATGGAAATGTAGTTCAACCAATATGACTTCATCATGGTTGCATCTGCACAATTCAGCATGAATCATGTGATCCTTGCCAAAACCATTGAGGACATGGGAA

IRGM_human     3133 AGAAGTTCTACATTGTCTGGACCAAGCTAGACATGGACCTCAGCACAGGTGCCCTCCCAGAAGTGCAGCTACTGCAGATCAGAGAAAATGTCCTGGAAAA
IRGM_chimp     3144 AGAAGTTCTACATTGTCTGGACCAAGCTGGACCTGGACCTCAGCACAGGTGCCCTCCCAGAAGTGCAGCTACTGCAGATCAGAGAAAATGTCCTGGAAAA
IRGM_orangutan3006 AGAAGTTCTACATTGTCTGGACCAAGCTGGACATGGACCTCAGCACAGGTGCCCTCCCAGAAGTGCAGCTACTGCAGATCAGAGAAAACGTCCTGGAAAA
IRGM_macaque   1470 AGAAGTTCTACATTGTCTGGACCAAGCTGGACATGGACCTCAGCACAGGTGCCCTCCCAGAAGTGCAGCTACTGCGGATCAGAGAAAATGTCCTGGAAAA
IRGM_marmoset 1458 AGAAGTTCTACATTGTCTGGACCAAGCTGGACATGGATCTCAGCACAGGTGCCCTCCCAGAAGTGCAGCTACTGTAAATCAGAGAAAATGTCCTGGAAAG

IRGM_human     3233 TCTCCAGAAGGA
IRGM_chimp     3244 TCTCCAGAAGGA
IRGM_orangutan3106 TCTCCAGAAGGA
IRGM_macaque   1570 TCTCCAGAAGGA
IRGM_marmoset 1558 TCTCCAGAGGGA
```
