# Supplementary material for: Death and Resurrection of the Human IRGM Gene
Source: PLoS Genet. 2009 Mar 6;5(3):e1000403. doi: 10.1371/journal.pgen.1000403 (PMC2644816; doi:10.1371/journal.pgen.1000403)
Supplement: Text S1 — Supplementary note: Death and resurrection of the human IRGM gene. (0.61 MB PDF) [file pgen.1000403.s007.pdf]

# Supplementary Note

## Death and resurrection of the human *IRGM* gene

**Cemalettin Bekpen[1,2], Tomas Marques-Bonet[1,3], Can Alkan[1,2], Francesca Antonacci[1], Bruna Leogrande[4], Mario Ventura[4], Jeffrey M. Kidd[1], Priscillia Siswara[1], Jonathan C. Howard[5], Evan E. Eichler[1,2]**

## Table of Contents

**I. Detailed Materials and Methods** .......... **2**

a) Expression Analyses .......... 2

b) Sequence Analyses .......... 2

c) FISH .......... 2

Supplementary Note Table 1. Proportion of codons assigned to neutral or purifying selection .......... 4

Supplementary Note Table 2. Log likelihood values and parameters estimates under different models of evolution .......... 4

Supplementary Note Table 3. Likelihood ratio statistics for hypothesis testing .......... 5

**II. *IRGM* Gene Structure and Organization** .......... **5**

Supplementary Note Figure 1. Synteny relationships among the human, macaque, dog and mouse *IRG* genes .......... 8

**III. Human Structural Polymorphism 5' Upstream of *IRGM*** .......... **8**

**References** .......... **9**

Supplementary Note Figure 2. Alignment of human *IRGM* splice variants .......... 10

Supplementary Note Figure 3. Nucleotide alignment of mammalian *IRGM* genes .......... 11

Supplementary Note Figure 4. Amino acid alignment of the *IRGM* proteins (full length) .......... 13

Supplementary Note Figure 5. Nucleotide alignment of Old World monkey *IRGM* genes .......... 14

Supplementary Note Figure 6. Nucleotide alignment of New World monkey *IRGM* genes .......... 15

Supplementary Note Table 4. *Microcebus murinus* BAC clones .......... 16

Supplementary Note Table 5. *Lemur catta* BAC library and FISH .......... 17

Supplementary Note Table 6. Primate DNA sample IDs and genotyping results .......... 18

Supplementary Note Table 7. PCR assays .......... 20

## I. Detailed Materials and Methods

### a) Expression Analyses

**RT-PCR.** cDNA was prepared using the Advantage RT PCR kit (Clontech) according to manufacturer instructions. mRNA, used for cDNA preparation, was extracted (Oligotex mRNA isolation kit, Qiagen) from total RNA prepared (RNA easy total RNA isolation kit, Qiagen) from primate tissues Ptr (chimp), Rh (macaque), Cja (marmoset) and Hs (human) (Supp. Note Table 6). UBE1 and GAPDH were used as positive controls (Supp. Note Table 7).

**Real-Time PCR.** *IRGM* splice variants were detected by a quantitative PCR assay using the LightCycler SYBR Green System (Roche) with primers *IRGM* (b)-(c)-(d) and *IRGM* (all) primers. cDNA was synthesized using mRNA prepared from lymphoblast cell lines. The amount of measured transcripts was normalized to the amount of the GAPDH and UBE1 transcript (Supp. Note Table 7).

**5' RACE PCR.** Single-stranded cDNA was prepared using a rapid PCR purification kit (Roche). The terminal deoxynucleotidyl transferase reaction was prepared as follows: 16.5µl cDNA, 5µl TdT + Reaction buffer (Amersham), 2.5µl dCTP (2mM) were incubated for 3 minutes at 94°C, 1µl of Tdt was added and incubated for 15 minutes at 37°C, followed by inactivation step for 5 minutes at 65°C. PCR was performed on the (cDNA+polyC) using the primer *5'Anc* and *IRGM-rGMS*. PCR products were purified using the rapid PCR purification kit and a second round nested PCR was performed using the primers *UAP* and *IRGMr1* (Supp. Note Table 7). A 1.7 kb PCR product (1.65 kb) was cloned to PGEM-T easy and insert sequences were determined by sequencing.

### b) Sequence Analyses

Whole genome shotgun sequence data from chimpanzee (*Pan troglodytes*), gorilla (*Gorilla Gorilla*), orangutan (*Pongo pygmaeus*), rhesus macaque (*Macaca mulatta*), marmoset *(Callithrix jacchus*), baboon (*Papio hamadryas*) and gray mouse lemur (*Microcebus murinus*) were retrieved from NCBI Trace Archive (http://www.ncbi.nlm.nih.gov/Traces/trace.cgi?). We searched for similarities of the human *IRGM* and its 5'-upstream sequence using human *IRGM* as BLAST query (parameters: -v 500 –b 500 –e 1e-10)[1]. We retrieved all WGS reads that were reported to contain *IRGM*-like sequences and constructed local sequence assemblies using PHRAP (default parameters, high stringency) (http://www.phrap.org). Repetitive elements were annotated using RepeatMasker software (http://www.repeatmasker.org).

### c) FISH

Metaphase spreads were obtained from lymphoblast or fibroblast cell lines from human (*Homo sapiens*), rhesus macaque (*Macaca mulatta*), marmoset (*Calithrix jacchus*) and lemur (*Lemur catta*). FISH was performed using genomic clones carrying the *IRGM* gene: human fosmid WIBR2-3607H18 and lemur BACs LB2-61D22, LB2-77B23 and LB-61A22. Probes were directly labeled by nick translation with Cy3-dUTP (Perkin-Elmer). Each hybridization used 300 ng of labeled probe, 5 mg Cot-1 DNA (Roche) and 3 mg sonicated salmon sperm DNA at 37$^{o}$C

in 10 ml 2xSSC/50% formamide/10% dextran sulfate, followed by three posthybridization washes at 60°C in 0.1 x SSC. Nuclei were stained with 4-diamidino-2-phenylindole (DAPI) and digital images obtained using a Leica DMRXA2 epifluorescence microscope equipped with a cooled CCD camera (Princeton Instruments). Cy3-dUTP and DAPI fluorescence signals, detected with specific filters, were recorded separately as gray scale images. Pseudocoloring and merging of images were performed using Adobe Photoshop™ software.

**d) Genomic Library Hybridization**

Lemur BACs were obtained using a PCR product derived from *M. murinus* genomic DNA as probe to screen genomic BAC libraries. The LBNL-2 lemur genomic library is 6.1-fold redundant (CHORI Resources: LBNL-2 Lemur BAC Library [http://gsd.jgi-psf.org/cheng/LB2]). The probe was hybridized to high-density filters of *L. catta* (LBNL-2) and *M. murinus* (Chori-257) BAC libraries according to a published protocol from Pieter De Jong (CHORI Resources: Hybridization of High Density Filters [http://bacpac.chori.org/highdensity.htm]). Images were analyzed with ArrayVision Ver6.0™ (Imaging Research Inc., Linton, UK).

**e) Phylogenetic Analyses**

All multiple sequence alignments were generated using ClustalW[2,3]. We constructed neighbor-joining phylogenetic trees (MEGA 3.1)[4]. To analyze the evolution of *IRGM*'s coding region across the phylogeny, we first retrieved the orthologous and paralogous sequences for dog and gray mouse lemur (*Microcebus murinus*) to be used as outgroups. All alignments were manually curated to ensure an open reading frame. We compared the ratio ($\omega = d_N/d_S$) of $d_N$ (non-synonymous substitutions per non-synonymous site) and $d_S$ (synonymous substitutions per synonymous site) using maximum likelihood methods (PAML)[5]. We divided our species into three groups according to their evolutionary history: Group 1 was composed of species that contain the ERV9 insertion (human (Hs), chimpanzee (Ptr), orangutan (Ppy) and gorilla (Ggo)); Group 2 was composed of species that do not possess an ERV9 integration and the open reading frame (ORF) has stop codons ( rhesus macaque (Rh), baboon (Pha) and marmoset (Cja)); and finally, Group 3 was formed by outgroup *IRGM* loci from dog and gray mouse lemur.

We then independently applied to every group a codon-substitution site model analysis[6] in which $\omega$ is allowed to vary among codons across the sequences. Using a Bayesian approach, every codon is assigned to a conserved ($\omega<1$) or to a neutral ($\omega=1$) category and the proportion of codons under neutral evolution or purifying selection are estimated. The results show that only Group 1 (Hs, Ptr, Ppy and Ggo) and Group 3 (dog and gray mouse lemur (*Microcebus murinus*, Mmu) have the majority of sites under negative constraint in contrast to Group 2 where the majority of codons were assigned to a neutral category (Supp. Note Table 1). We statistically rejected the null hypothesis of a codon-substitution site models with positive selection that might have explained this excess of neutral codons in Group 2 (M1 vs M2, $P=0.74$, and M7 vs M8, $P=0.75$).

Next, we applied a codon-substitution branch model[7] to estimate evolutionary pressures at different times during the evolution of this gene family. We constructed different codon-substitution models to provide a statistical framework for gene evolution (Supp. Note Table 2).

Formal tests comparing the likelihoods of different models under different ω values for different groups were applied using a Likelihood Ratio Test (LRT) (Supp. Note Table 3).

**Supplementary Note Table 1. Proportion of codons assigned to neutral or purifying selection**

| Parameter | Group 1 | | Group 2 | | Group 3 | |
|---|---|---|---|---|---|---|
| ω | 0.5763 | 1 | 0 | 1 | 0.1427 | 1 |
| Percentage of codons | 100.00% | 0.00% | 27.73% | 72.27% | 65.27% | 34.73% |

A codon site-class model (CODEML) analysis[6] was applied to each group to estimate the fraction of codons that have been subjected to negative selection within each group. The majority of codons in Group 1 (Hs, Ptr, Ppy and Ggo) and Group 3 (dog and gray mouse lemur (*Microcebus murinus*, Mmu) are consistent with a model of negative constraints whereas the majority of codons in Group 2 are consistent with a neutral model of evolution (ω=1).

Twice the difference in the log-likelihoods of related models can be fit to a Chi-square distribution to obtain significance for the alternate model. First, we used a model in which we set the rates of the evolution of the branches in Group 2 to a model of purifying selection (Group 2, ω=0.5). The difference between the model of purifying selection (Group 2, ω=0.5) against a codon-substitution free model based on the three groups (Group 2, ω=0.91) was significantly different (P=0.013)($X^2$=6.17, df=1), providing additional support that Group 2 is evolving under a neutral model of selection. Testing a neutral model for Group 2 (Group 2, ω=1) was statistically indistinguishable (P=0.75) from our "three-groups free model". Next, we used a model in which Group 3 phylogeny was set to a neutrally-evolving codon-substitution model (Group 3, ω=1). This model was statistically rejected (P=6.09$E^{-12}$) compared to a three-groups free model (Group3, ω=0.39) indicating purifying selection. Finally, we found that a neutral model of codon substitution for Group 1 (Group 1, ω=1) was indistinguishable (P=0.22) from our three-groups free model (Group 1, ω=0.61). Short branch length and the limited number of closely related species limit the power to detect selection within these lineages.

**Supplementary Note Table 2. Log likelihood values and parameters estimates under different models of evolution**

| # | Model | **l** | **NP** |
|---|---|---|---|
| 1 | Free Branch Model | -2488.90 | 47 |
| 2 | 3 Groups (three-Groups free model) | -2497.75 | 27 |
| 3 | 3 Groups with Group 2 set to negative constraints (ω=0.5) | -2500.84 | 26 |
| 4 | 3 Groups with Group 1 set to neutral evolution (ω=1) | -2498.49 | 26 |
| 5 | 3 Groups with Group 3 set to neutral evolution (ω=1) | -2521.40 | 26 |

We constructed five codon-substitution branch models to test different hypotheses regarding the evolution of *IRGM* coding sequence. **l**=log likelihood values of the models; **NP=**number of parameters estimated in the models

**Supplementary Note Table 3. Likelihood ratio statistics for hypothesis testing**

| Hypothesis Tested | Models Compared | 2*Δ l | diff NP | P-value (chi-square) | Result |
|---|---|---|---|---|---|
| Can we group our tree into three groups?<br>Ho: 3 groups<br>Ha: Free | 1 vs 2 | 17.71 | 20 | 0.6068 | We can group the tree into three groups |
| Is Group 2 conserved?<br>Ho: ω=0.5<br>Ha: ω =0.91 | 2 vs 3 | 6.17 | 1 | **0.0130** | Group 2 is under neutral evolution, since the constrained model is statistically different |
| Is Group 1 evolving neutrally?<br>Ho: ω =1<br>Ha: ω =0.61 | 2 vs 4 | 1.47 | 1 | 0.2257 | Group 1 is not statistically different than a neutral model |
| Is Group 3 evolving neutrally ?<br>Ho: ω =1<br>Ha: ω =0.39 | 2 vs 5 | 47.30 | 1 | **$6.09E^{-12}$** | Group 3 is not under neutral evolution since the neutral model is statistically different |
| Is the excess of neutral sites in Group 2 due to positive selection? | M1 vs M2 (Codon-substitution site models) | 0.6 | 2 | **0.74** | Excess of codons under neutral evolution in Group 2 cannot be explained by positive selection |

Several likelihood ratio tests were performed to contrast different hypotheses. **2*Δ l**=Twice the difference in likelihoods of the null (Ho) versus the alternative (Ha) hypothesis. This value can fit in a chi-square distribution to assess the significance. **diff NP**=Differences in the number of parameters estimated in the models compared. **P-value (chi-square)**=P-value of the Chi-square value in a distribution with as many degrees of freedom as differences in the parameters estimated.

The combined results of this analysis suggest that different evolutionary pressures have been acting across the phylogeny of this gene in mammals. According to the codon-substitution site model analysis and the results for the codon-substitution branch-free model analysis, *IRGM* in dog and prosimian have been under strong purifying selective constraint. Conversely, we find that the primate branch leading to marmoset, macaque and baboon was evolving neutrally, suggesting pseudogenization at the evolutionary time corresponding to the divergence of prosimian and anthropoid lineages. However, we cannot rule out the possibility that the gene became pseudogenized independently in New World and Old World monkey lineages.

We note that after the ERV9 integration ω tends to be reduced in almost all species of apes (human, chimp and orangutan), and the codon-substitution site model analysis clearly assigns the majority of codons within this group to an $\omega<1$. In addition, almost all values of ω estimated for these branches by maximum likelihood are similarly less than one. These data suggest that *IRGM* in human, chimp and orangutan may be under weak negative selection possibly due to a newly acquired or recovered function.

## II. *IRGM* Gene Structure and Organization

Detailed database and 5' RACE PCR analysis revealed that the human *IRGM* promoter region, including the beginning of 5' UTR region, corresponds to the ERV9 retroviral integration that

emerged in the common ancestor of humans and apes but after the divergence from the Old World monkey lineages (Fig. 1 and Fig. S4). The encoded human *IRGM* protein is truncated at the C-terminus when compared to the mouse. Sequencing of multiple partial- and full-length cDNA reveals multiple alternative splice forms of *IRGM*. Five different splice forms have been identified: *IRGM* (a), (b), (c), (d), (e). The longest open reading frame is *IRGM* (b) (Supp. Note Fig. 2)[8]. None of the splice variants are predicted to produce a protein in excess of 25 kD.

Analysis of gray mouse lemur (*Microcebus murinus)* whole genome shotgun sequence reveals multiple copies of the *IRG* gene family (Fig. 1). To verify the database search, we screened a mouse lemur BAC library using an *IRGM* PCR product amplified from mouse lemur DNA as a hybridization probe. Genomic colony hybridizations identified 25 different BAC clones and partial sequencing of these BAC clones recovered at least three *IRGM* sequences belonging to the GMS-type classification: *IRGM7*, *IRGM9* and one pseudogene *IRGM8*. *IRGM8* and *9* are closely related to each other and are likely the result of recent tandem duplication (Fig. 1, Supp. Note Fig. 3 and Table 4). Other members of the GKS type *IRG* gene family are also present in this species suggesting that similar to dog, the mouse lemur has a functional *IRG* protein family (data not shown). Gray mouse lemur (*M. murinus*) *IRGM9* encodes a putative 47 kDa protein including conserved motifs at the carboxyl-terminus that are specific to mouse *IRG* proteins. *IRGM7* also encodes a predicted 47 kDa protein but has non-canonical substitutions in G domain that disrupts the G1 motif (GXXXXGMS > GXXXXDMS) of P loop GTPases. (Fig. S1 and Supp. Note Fig. 4)[9]. *IRGM8* is likely a pseudogene because of a substitution generating a stop codon within the G domain and an additional frameshift mutation at the C terminus. Our sequence analysis confirms that none of the mouse lemur *IRGM* genes contain an ERV9 retroviral element. Fluorescence in situ hybridization (FISH) and sequence analyses using a BAC library from a second prosimian outgroup (*Lemur catta)* confirm multiple copies of the *IRGM* gene family with at least two tandem duplications and possibly three interchromosomal duplications in a region syntenic to human *IRGM* (Fig. 2 and Supp. Note Table 5). We note that an important structural difference between anthropoids and prosimian *IRGM* genes is the presence of an Alu $S_c$ retroposon insertion immediately after the splicing acceptor that disrupts the ORF of *IRGM* in all anthropoid lineages. It is possible that this Alu insertion within the ORF of *IRGM* may have contributed to the non-functionalization of the remaining *IRGM* gene.

Sequence analysis of the single copy marmoset *IRGM* locus suggests a pseudogene. The ORF is truncated at the C terminus and has two stop codons within the canonical ORF (Supp. Note Fig. 3). Similar to the gray mouse lemur, there is no ERV9 retroviral element in the promoter region of the marmoset *IRGM*, but there is an $AluS_c$ repeat element mapping in the ORF of *IRGM* (Figs. 1, S2 and S4). Only a single copy of *IRGM* could be detected based both on sequence and FISH analysis (Fig. 2). Sequence analysis from DNA from four diverse species representing New World monkeys showed that stop codons were shared in all species tested (Supp. Note Fig. 5). This suggests that the stop codons arose and were fixed early in an ancestral species.

The rhesus macaque (*Macaca mulatta*) genome also contains a single truncated *IRGM* sequence mapping to chromosome 6. Similar to marmoset, the orthologous sequence does not maintain an open reading frame due to frameshift mutation, lacks an ERV9 element and possesses an $AluS_c$ sequence after the splicing acceptor (Figs. 1, S2 and S4). The overall baboon (*Papio hamadryas*, Pha) *IRGM* structure is identical to macaque, including identical stop codons suggesting that the

pseudogene has persisted more than 9 million years within this lineage of primate evolution (estimated divergence time between baboon and macaque). Sequencing of the *IRGM* locus from five unrelated macaques, five unrelated baboons, and nine species from Old World monkeys confirmed that the frameshift mutation and stop codon are conserved and the *IRGM* ORF is disrupted in the phylogeny of Old World monkeys (Fig. S3, Supp. Note Fig. 3 and 6).

Sequencing of the *IRGM* locus in all great apes reveals a restored ORF with the exception of orangutan where the stop codon is polymorphic. In addition, the promoter and 5' UTR regions of orangutan *IRGM* are identical to chimp and human with the exception of two deletions (15 and 118 bp in length) within the ERV9 element (Fig. S4). Analysis of the orangutan genome revealed again a single copy of *IRGM*, truncated at the C-terminus, as in human and the earlier anthropoids. One of three gibbon species and three of eight orangutan individuals were heterozygous for a C->T transition at nucleotide position 150 relative to the start codon resulting in premature termination (Figs. 1, Supp. Note Fig. 3 and 5). Thus the C-terminally truncated *IRGM* gene in orangutan is polymorphic, with one allele putatively producing a 20 kD protein. Human and chimpanzee *IRGM* share 97% identity at the nucleotide level. Gorilla *IRGM* gene organization is similar to human, chimp and orangutan including Alu and ERV9 retroviral element. Sequence analysis from DNA from multiple unrelated humans (n=5), chimp (n=5) and gorilla (n=3) showed that the truncated *IRGM* has a complete ORF in all three species (Supp. Note Table 7).

Summary: In contrast to mouse, dog and prosimian (*M. murinus* and *L. catta*), which show evidence of multiple members of the *IRG* gene family, our analysis suggests that anthropoids have only a single *IRGM* gene that is truncated at C terminus, relative to a typical *IRG* gene product, and encoding the G domain of a GMS type *IRG* protein (Figs. 1, 2 and S3). Our analysis of *IRGM* ORF indicates that the single copy gene is pseudogenized in marmoset, macaque and baboon perhaps due to the Alu $S_c$ repeat integration immediately after the splicing acceptor that disrupts the ORF of the sole remaining *IRGM* gene (Figs. 5 and S2). By subsequent integration of the ERV9 element, the *IRGM* gene appears to have regained its ORF in the hominoids (Figs. 1, 2 and Supp. Note Fig. 6). No *IRG* proteins were detected in anthropoids except *IRGC* suggesting that *IRG* proteins disappeared from the primate lineage after the divergence of the anthropoids from prosimians (50 mya) (Rohde C. et al., Manuscript in preparation). We note that comparative synteny maps across various mammalian species (UCSC genome browser) suggest that *IRGM* locus corresponds to a breakpoint of synteny between primate and murine genomes. Interestingly, the dog *IRGM* cluster appears to have evolved at a non-orthologous location within the Canfam2 assembly (Supp. Note Fig. 1).

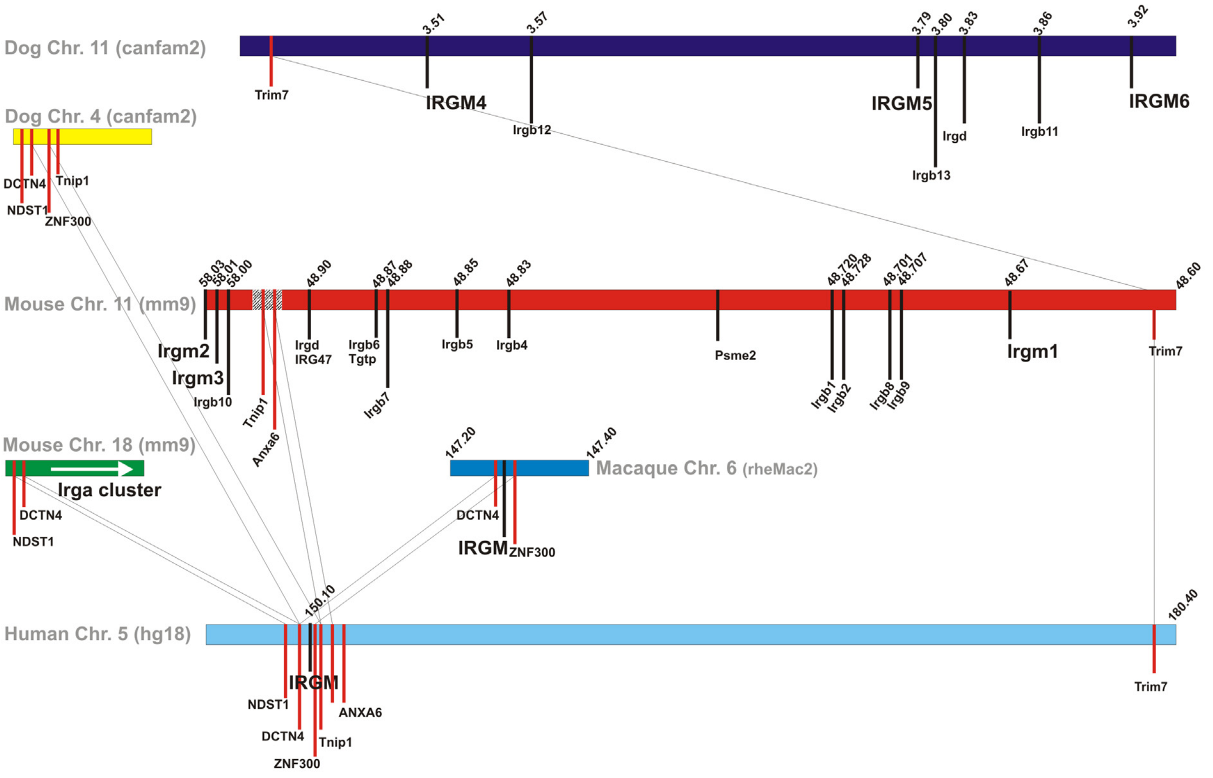


**Supplementary Note Figure 1. Synteny relationships among the human, macaque, dog and mouse *IRG* genes**
Complex synteny relationships among human chr. 5, macaque chr. 6, dog chrs. 11 and 4, and mouse chrs. 11 and 18 are compared with respect to *IRGM* and flanking genes. Distances from the centromere in megabases are indicated based on the genome assembly (brackets). Syntenic markers are given in red color; shaded boxes indicate chromosome gap regions in the assembly.

## III. Human Structural Polymorphism 5' Upstream of *IRGM*

Analysis of fosmid libraries from nine individuals identified a deletion near the human *IRGM* gene[11]. Examination of a high quality sequence from a clone spanning the variant haplotype (AC207974, from NA18956 [ABC9]) confirms a 20.1 kbp deletion 2.82 kb 5' from the *IRGM* start site, as well as a 1.95 kb upstream of the ERV9 sequence (Fig. S5). Furthermore, it has recently been shown that this deleted region is polymorphic among humans with 7/18 interrogated chromosomes carrying the deletion configuration (Figs. S5 and S6)[11]. The polymorphic 20.1 kb region contains an LTR and a number of repeat (LINE) sequence (Fig. S6). Because the deletion is only 1.95 kb upstream of the ERV9 retroviral element, we asked whether this structurally polymorphic region has any effect on mRNA expression of human *IRGM*. To find possible alterations in the expression profile, we used structurally characterized lymphoblast cell lines GM18555, GM15510 and GM18507, homozygous for deletion (D/D), heterozygous for insertion (I/D) and homozygous for insertion (I/I), respectively (Fig. S5). Our findings suggest that structural variation at the 5' upstream of *IRGM* has an effect on the level of mRNA expression as well as a proportion of the alternative splice versions of human *IRGM* (Fig. S5). Using *IRGM* gene specific primers that amplify all splice variants (Supp. Note Table 7), *IRGM* is detected to be highly expressed in GM18555 (D/D), at a moderate level in GM18507 (I/I) and

low level in GM15510 (I/D) when compared to one another. We verified that *IRGM* (c) expression is higher in GM18507 (I/I). *IRGM* (d) is expressed at low level in GM18555 (D/D). Our findings suggest a correlation between the insertion polymorphism and expression. We find that *IRGM* (b) transcript is down-regulated and alternative spliced versions of *IRGM* are up-regulated in the presence of the insertion (Fig. S5).

**References**


1. S. F. Altschul, W. Gish, W. Miller et al., *J Mol Biol* **215** (3), 403 (1990).
2. J. D. Thompson, D. G. Higgins, and T. J. Gibson, *Nucleic acids research* **22** (22), 4673 (1994).
3. R. Chenna, H. Sugawara, T. Koike et al., *Nucleic acids research* **31** (13), 3497 (2003).
4. S. Kumar, K. Tamura, and M. Nei, *Comput Appl Biosci* **10** (2), 189 (1994).
5. Z. Yang, *Molecular biology and evolution* **24** (8), 1586 (2007).
6. Z. Yang, *Journal of molecular evolution* **51** (5), 423 (2000).
7. Z. Yang, *Molecular biology and evolution* **15** (5), 568 (1998).
8. C. Bekpen, J. P. Hunn, C. Rohde et al., *Genome Biol* **6** (11), R92 (2005).
9. D. D. Leipe, Y. I. Wolf, E. V. Koonin et al., *J Mol Biol* **317** (1), 41 (2002).
10. S. B. Singh, A. S. Davis, G. A. Taylor et al., *Science (New York, N.Y* **313** (5792), 1438 (2006).
11. J. M. Kidd, G. M. Cooper, W. F. Donahue et al., *Nature* **453** (7191), 56 (2008).
12. M. F. Cardone, M. Ventura, S. Tempesta et al., *Chromosoma* **111** (5), 348 (2002).

**Supplementary Note Figure 2. Alignment of human *IRGM* splice variants**

Alignment of *IRGM* splice variants using ClustalW (http://www.ebi.ac.uk/Tools/clustalw2/index.html). Boxshade denotes conserved exons among the different splice variants: *IRGM* (a), (b), (c), (d), (e). *IRGM* (a) shows no evidence of splicing and *IRGM* (c), (d) and (e) are subjected to nonsense mediated mRNA decay (NMD) because they contain a premature stop codon. *IRGM* (a) and (b) are thought to represent the only functional splice form.

```
IRGM(a)    1 ATGAATGTTGAGAAAGCCTCAGCAGATGGGAACTTGCCAGAGGTGATCTCTAACATCAAGGAGACTCTGAAGATAGTGTCCAGGACACCAGTTAACATCA
IRGM(b)    1 ATGAATGTTGAGAAAGCCTCAGCAGATGGGAACTTGCCAGAGGTGATCTCTAACATCAAGGAGACTCTGAAGATAGTGTCCAGGACACCAGTTAACATCA
IRGM(c)    1 ATGAATGTTGAGAAAGCCTCAGCAGATGGGAACTTGCCAGAGGTGATCTCTAACATCAAGGAGACTCTGAAGATAGTGTCCAGGACACCAGTTAACATCA
IRGM(d)    1 ATGAATGTTGAGAAAGCCTCAGCAGATGGGAACTTGCCAGAGGTGATCTCTAACATCAAGGAGACTCTGAAGATAGTGTCCAGGACACCAGTTAACATCA
IRGM(e)    1 ATGAATGTTGAGAAAGCCTCAGCAGATGGGAACTTGCCAGAGGTGATCTCTAACATCAAGGAGACTCTGAAGATAGTGTCCAGGACACCAGTTAACATCA

IRGM(a)  101 CTATGGCAGGGGACTCTGGCAATGGGATGTCCACCTTCATCAGTGCCCTTCGAAACACAGGACATGAGGGTAAGGCCTCACCTCCTACTGAGCTGGTAAA
IRGM(b)  101 CTATGGCAGGGGACTCTGGCAATGGGATGTCCACCTTCATCAGTGCCCTTCGAAACACAGGACATGAGGGTAAGGCCTCACCTCCTACTGAGCTGGTAAA
IRGM(c)  101 CTATGGCAGGGGACTCTGGCAATGGGATGTCCACCTTCATCAGTGCCCTTCGAAACACAGGACATGAGGGTAAGGCCTCACCTCCTACTGAGCTGGTAAA
IRGM(d)  101 CTATGGCAGGGGACTCTGGCAATGGGATGTCCACCTTCATCAGTGCCCTTCGAAACACAGGACATGAGGGTAAGGCCTCACCTCCTACTGAGCTGGTAAA
IRGM(e)  101 CTATGGCAGGGGACTCTGGCAATGGGATGTCCACCTTCATCAGTGCCCTTCGAAACACAGGACATGAGGGTAAGGCCTCACCTCCTACTGAGCTGGTAAA

IRGM(a)  201 AGCTACCCAAAGATGTGCCTCCTATTTCTCTTCCCACTTTTCAAATGTGGTGTTGTGGGACCTGCCTGGCACAGGGTCTGCCACCACAACCCTGGAGAAC
IRGM(b)  201 AGCTACCCAAAGATGTGCCTCCTATTTCTCTTCCCACTTTTCAAATGTGGTGTTGTGGGACCTGCCTGGCACAGGGTCTGCCACCACAACCCTGGAGAAC
IRGM(c)  201 AGCTACCCAAAGATGTGCCTCCTATTTCTCTTCCCACTTTTCAAATGTGGTGTTGTGGGACCTGCCTGGCACAGGGTCTGCCACCACAACCCTGGAGAAC
IRGM(d)  201 AGCTACCCAAAGATGTGCCTCCTATTTCTCTTCCCACTTTTCAAATGTGGTGTTGTGGGACCTGCCTGGCACAGGGTCTGCCACCACAACCCTGGAGAAC
IRGM(e)  201 AGCTACCCAAAGATGTGCCTCCTATTTCTCTTCCCACTTTTCAAATGTGGTGTTGTGGGACCTGCCTGGCACAGGGTCTGCCACCACAACCCTGGAGAAC

IRGM(a)  301 TACCTGATGGAAATGCAGTTCAACCGGTATGACTTCATCATGGTTGCATCTGCACAATTCAGCATGAATCATGTGATGCTTGCCAAAACCGCTGAGGACA
IRGM(b)  301 TACCTGATGGAAATGCAGTTCAACCGGTATGACTTCATCATGGTTGCATCTGCACAATTCAGCATGAATCATGTGATGCTTGCCAAAACCGCTGAGGACA
IRGM(c)  301 TACCTGATGGAAATGCAGTTCAACCGGTATGACTTCATCATGGTTGCATCTGCACAATTCAGCATGAATCATGTGATGCTTGCCAAAACCGCTGAGGACA
IRGM(d)  301 TACCTGATGGAAATGCAGTTCAACCGGTATGACTTCATCATGGTTGCATCTGCACAATTCAGCATGAATCATGTGATGCTTGCCAAAACCGCTGAGGACA
IRGM(e)  301 TACCTGATGGAAATGCAGTTCAACCGGTATGACTTCATCATGGTTGCATCTGCACAATTCAGCATGAATCATGTGATGCTTGCCAAAACCGCTGAGGACA

IRGM(a)  401 TGGGAAAGAAGTTCTACATTGTCTGGACCAAGCTAGACATGGACCTCAGCACAGGTGCCCTCCCAGAAGTGCAGCTACTGCAGATCAGAGAAAATGTCCT
IRGM(b)  401 TGGGAAAGAAGTTCTACATTGTCTGGACCAAGCTAGACATGGACCTCAGCACAGGTGCCCTCCCAGAAGTGCAGCTACTGCAGATCAGAGAAAATGTCCT
IRGM(c)  401 TGGGAAAGAAGTTCTACATTGTCTGGACCAAGCTAGACATGGACCTCAGCACAGGTGCCCTCCCAGAAGTGCAGCTACTGCAGATCAGAGAAAATGTCCT
IRGM(d)  401 TGGGAAAGAAGTTCTACATTGTCTGGACCAAGCTAGACATGGACCTCAGCACAGGTGCCCTCCCAGAAGTGCAGCTACTGCAGATCAGAGAAAATGTCCT
IRGM(e)  401 TGGGAAAGAAGTTCTACATTGTCTGGACCAAGCTAGACATGGACCTCAGCACAGGTGCCCTCCCAGAAGTGCAGCTACTGCAGATCAGAGAAAATGTCCT

IRGM(a)  501 GGAAAATCTCCAGAAGGAGCGGGTATGTGAATACTAA---------------------------------------------------------------
IRGM(b)  501 GGAAAATCTCCAGAAGGAGCGG------------------------------------------------------------------------------
IRGM(c)  501 GGAAAATCTCCAGAAGGAGCGGAGCTCTAGAAACCAACAAGTGTACCCAAACCACAGCCACGTACCTCTTGCTTGCTGAAAGGATCCTGAGAGAAAGGCA
IRGM(d)  501 GGAAAATCTCCAGAAGGAGCGG------------------------------------------------------------------------------
IRGM(e)  501 GGAAAATCTCCAGAAGGAGCGGAGCTCTAGAAACCAACAAGTGTACCCAAACCACAGCCACGTACCTCTTGCTTGCTGAAAGGATCCTGAGAGAAAAGCA

IRGM(a)      ----------------------------------------------------------------------------------------------------
IRGM(b)  523 ---------------------------------------------------------CTGGCCTGCCATGAGAAATACCTCAAGAGTACTCCAGAGAATT
IRGM(c)  601 AGACAAAAAAATACAGATCAGCTCCGCAAGCCCTAGAAATAGCAAGGCCCAGCTCAGCTGGCCTGCCATGAGAAATACCTCAAGAGTACTCCAGAGAATT
IRGM(d)  523 ---------------------------------------------------------CTGGCCTGCCATGAGAAATACCTCAAGAGTACTCCAGAGAATT
IRGM(e)  601 AGACAAAGAAATACAGATCAGCTCCGCAAGCCCTAGAAATAGCAAGGCCCAGCTCAGCTGGCCTGCCATGAGAAATACCTCAAGAGTACTCCAGAGAATT

IRGM(a)      ----------------------------------------------------------------------------------------------------
IRGM(b)  566 CCACAAGGCCCAGAAATAT---------------------------------------------------------------------------------
IRGM(c)  701 CCACAAGGCCCAGAAATAT---------------------------------------------------------------------------------
IRGM(d)  566 CCACAAGGCCCAGAAATATAAATCTCTGCAGCTGACCATCACCTTGACAGACTTTTAAAATGGAGCACAATGAACCATCCCTTGTGACTCCTTTCAGTAT
IRGM(e)  701 CCACAAGGCCCAGAAATATAAATCTCTGCAGCTGACCATCACCTTGACAGACTTTTAAAATGGAGCACAATGAACCATCCCTTGTGACTCCTTTCAGTAT

IRGM(a)      ----------------------------------------------------------------------------------------------------
IRGM(b)  585 ----------------------------------------------------------------------------------------------------
IRGM(c)  720 ----------------------------------------------------------------------------------------------------
IRGM(d)  666 CTTATGATGGAAGGAAACTGTCCCCAAAATACATGACTGGGAGTTGTGAAGGTTACTCTTCCTGTCTAAAAGAAGAAAAGATACAATTTAAGAGTCATCA
IRGM(e)  801 CTTATGATGGAAGGAAACTGTCCCCAAAATACATGACTGGGAGTTGTGAGGGTTACTCTTCCTGTCTAAAAGAAGAAAAGATACAATTTAAGAGTCATCA

IRGM(a)      ----------------------------------------------------------------------------------------------------
IRGM(b)  585 ----------------------------------------------------------------------------------------------------
IRGM(c)  720 ----------------------------------------------------------------------------------------------------
IRGM(d)  762 CAAGAGTCAATTAAGAGGGTAAAGAAGTAGAACAAATGGATGATCCAACCATAGTAGATGAACTCACAAAAAAAGGATGGAAACAGGTCATCTCCAGAAG
IRGM(e)  897 CAAGAGTCAATTAAGAGGGTAAAGAAGTAGAACAAATGGATGATCCAAC-ATAGTAGATGAACTCACAAAAAAAGGATGGAAACAGGTCATCTC-AGAAG

IRGM(a)      ----------------------------------------------------------------------------------------------------
IRGM(b)  585 ----------------------------TCCTTCCAGAAGAAAACTATACGTAAATCTGCTCAGGATATTCAACAGCTAGCCAGTGCATATGACATATAC
IRGM(c)  720 ----------------------------TCCTTCCAGAAGAAAACTATACGTAAATCTGCTCAGGATATTCAACAGCTAGCCAGTGCATATGACATATAC
IRGM(d)  862 ACCAGTACCAGCCATATAGTGTTATCCGTCCTTCCAGAAGAAAACTATACGTAAATCTGCTCAGGATATTCAACAGCTAGCCAGTGCATATGACATATAC
IRGM(e)  995 ACCAGTACAG--CATATAGTGTTATCCGTCCTTCCAGAAGAAAACTATACGTAAATCTGCTCAGGATATTCAACAGCTAGCCAGTGCATATGACATATAC

IRGM(a)      ---------------------------------------------------------------------------
IRGM(b)  653 TGGGTACTTAAGAAACGTTTGTTAAATGAATCATGATTATAAAGAAAAAATAAATTAAAACTTATGTTTAGTTAA
IRGM(c)  788 TGGGTACTTAAGAAACGTTTGTTAAATGAATCATGATTATAAAGAAAAAATAAATTAAAACTTATGTTTAGTTAA
IRGM(d)  962 TGGGTACTTAAGAAACGTTTGTTAAATGAATCATGATTATAAAGAAAAAATAAATTAAAACTTATGTTTAGTTAA
IRGM(e) 1093 TGGGTACTTAAGAAACGTTTGTTAAATGAATCATGATTATAAAGAAAAAATAAATTAAAACTTATGTTTAGTTAA
```

**Supplementary Note Figure 3. Nucleotide alignment of mammalian *IRGM* genes**

Alignment of G-domain of all *IRGM* genes. Red highlighted sequences indicate the position of either a frameshift mutation or an in-frame stop codon. Species names are indicated as: Hs (*Homo sapiens*), Ptr (*Pan troglodytes*), Ggo (*Gorilla gorilla*), Ppy (*Pongo pygmaeus*), Hga (gibbon, *Hylobates gabriellae*), Rh (rhesus macaque, *Macaque mulatta*), Cja (*Callithrix jacchus*), Pph (*Papio hamadryas*). *IRGM*7, *IRGM*8, *IRGM*9 are (*Microcebus murinus*) paralogs, *IRGM*4, *IRGM*5, *IRGM*6 (Dog *IRGM* GMS type GTPases paralogs), and *Irgm*1, *Irgm*2, *Irgm*3 (Mouse *IRGM* GMS type GTPase paralogs). Species names, indicated as Ppy_IRGM_s and Hga_IRGM _s, show the presence of a pseudogene (C to T transition at the 150$^{th}$ nucleotide from the start codon. *IRGM*-encoding putative 20 kD protein indicated).

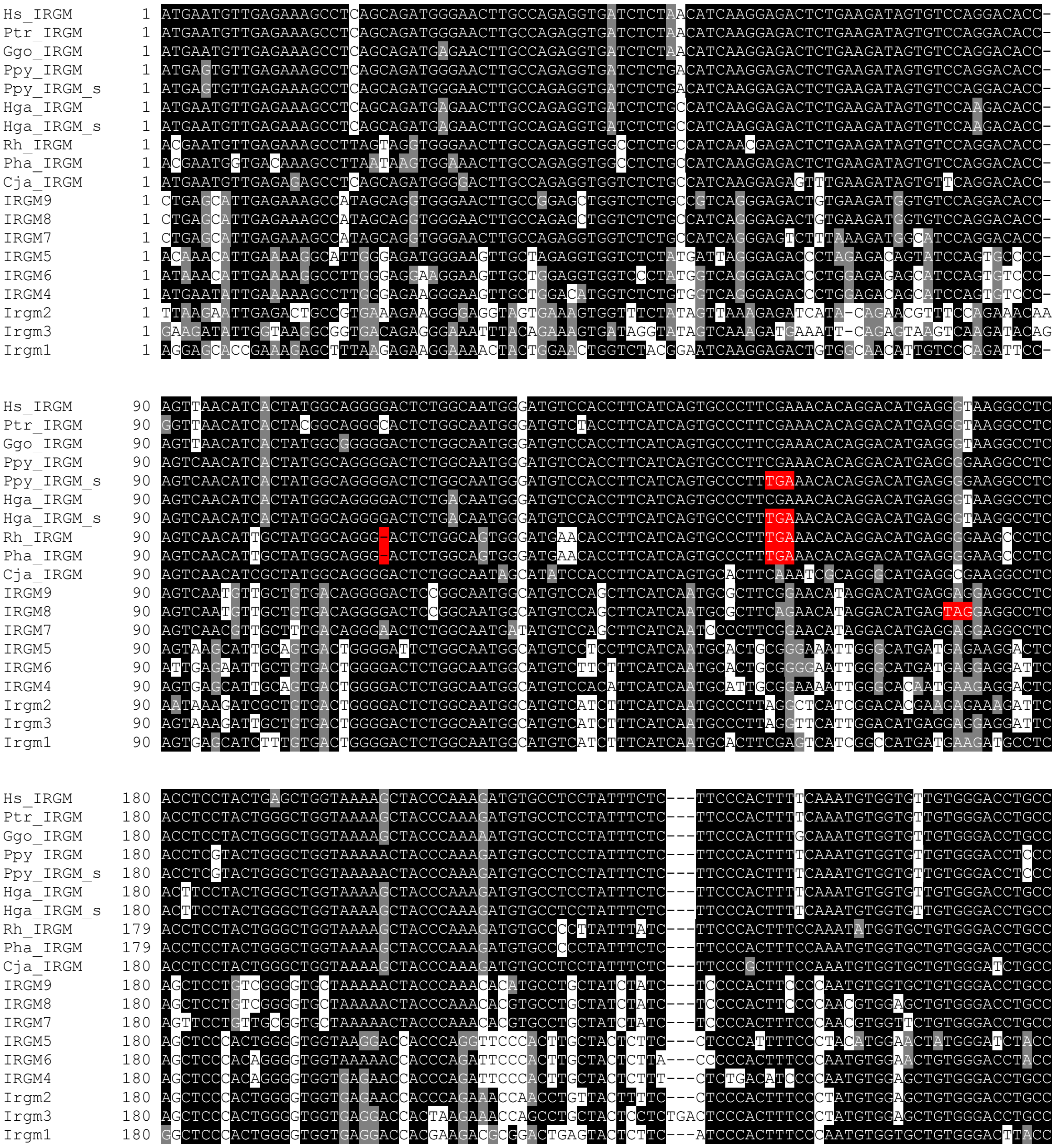

```
Hs_IRGM      267 TGGCACAGGGTCTGCCACCACAACCCTGGAGAACTACCTGATGGAAATGCAGTTCAACCGGTATGACTTC---ATCATGGTTGCATCTGC
Ptr_IRGM     267 TGGCACAGGGTCTGCCACCAAAACCCTGGAGAACTACCTGATGGAAATGCAGTTCAACCGGTATGACTTC---ATCATGGTTGCATCTGC
Ggo_IRGM     267 TGGCACTGGGTTTGCCACCAAAACCCTGGAGAACTACCTGATGGAAATGCAGTTCAACCGGTATGACTTC---ATCATGGTTGCATCTGC
Ppy_IRGM     267 TGGCACAGGGTCTGCCACGAAAACCCTGGAGAACTACCTGATGGAAATGCAGTTCAACCAGTGTGACTTC---ATCATGGTTGCATCTGC
Ppy_IRGM_s   267 TGGCACAGGGTCTGCCACGAAAACCCTGGAGAACTACCTGATGGAAATGCAGTTCAACCAGTGTGACTTC---ATCATGGTTGCATCTGC
Hga_IRGM     267 TGACACAGGGTCTGCCACCAAAACCTTGGAGAATTACCTGATGGAAATGCAGTTCAACCAGTATGCCTTC---ATCATGGTTGCATCTGC
Hga_IRGM_s   267 TGACACAGGGTCTGCCACCAAAACCTTGGAGAATTACCTGATGGAAATGCAGTTCAACCAGTATGCCTTC---ATCATGGTTGCATCTGC
Rh_IRGM      266 TGGCACAGGGTCTGCCACCAAAACCCTGGAGAACTACCTCATGGAAATGCAGTTCAACCAGTATGACTTC---ATCATGGTTGCATCTGC
Pha_IRGM     266 TGGCACAGGGTCTGCCACCAAAACCCTGGAGAACTACCTCATGGAAATGCAGTTCAACCAGTATGACTTC---ATCATGGTTGCATCTGC
Cja_IRGM     267 TGGAGCAGGGTCTGCCACCAAAACTCTGGAGAACTACCTGATGGAAATGTAGTTCAACCAATATGACTTC---ATCATGGTTGCATCTGC
IRGM9        267 TGGCACAGTGTCCGCCGCCCAAAGCCTGGAGAACTATGCGACGGAAATGCAGTTCAGCCGATATGACTTCTTCATCATCATCGCGTCTGA
IRGM8        267 TGGCACAGAGTGTGCCGCCCAAAGCCTGGAGAACTATGCGACGGAAATGCAGTTCAGCCGATATGACTTCTTCATCATCATCGCGTCTGA
IRGM7        267 TGGCACAGAGTGTGCCGCCCAAAGCCTGGAGAACTATTCTATGGAAATGCAGTTCAGCCGATATGACTTCTTCATCATCATCGTGTCTGA
IRGM5        267 TGGAACAGGGACAGGCACCCAAAGCTTGGAGAACTATCTGGAGAAGATACATTTTAGCCAGTATGACCTTTTCATCATCATTGCATCTGA
IRGM6        267 TGGAACAGGGGCAGGCACCCAAAGTCTGGAGAACTACCTGGAGGAGATGAAATTTAGCTGGTATGACCTCTTCATCATTATTGCATCTGA
IRGM4        267 TGGCACAGGCGCTGCCACCCAAAACCTAGAGACATATCTGGAGGAAATGCAGTTTAGCAAGTATGACCTCTTCATCATCATTGCATCTGA
Irgm2        267 TGGCTTAGGGGCCACAGCCCAGAGTGTGGAGAGCTACCTGGAAGAGATGCAGATCAGCATATACGACCTTATCATCATCGTAGCTTCTGA
Irgm3        270 TGGCTTAGGGGCCACAGCCCAGAGTGTGGAGAGCTACCTGGAGGAGATGCAGATCAGCACATTTGACCTTATCATCATCGTAGCTTCTGA
Irgm1        267 TGGATTGGGGGCCACAGCCCAAACCGTAGAGGACTATGTGGAAGAGATGAAATTTAGCACATGTGACTTATTCATCATCATTGCCTCTGA

Hs_IRGM      354 ACAATTCAGCATGAATCATGTGATGCTTGCCAAAACCGCTGAGGACATGGGAAAGAAGTTCTACATTGTCTGGACCAAGCTAGACATGGA
Ptr_IRGM     354 ACAATTCAGCATGAATCATGTGATGCTTGCCAAAACCGCTGAGGACATGGGAAAGAAGTTCTACATTGTCTGGACCAAGCTGGACCTGGA
Ggo_IRGM     354 ACAATTCAGCATGAATCATGTGATGCTTGCCAAAACCCCTGAGGACATGGGAAAGAAGTTCTACATTGTCTGGACCAAGCTGGACATGGA
Ppy_IRGM     354 ACAATTCAGCATGAATCAGGTGATGCTTGCCAAAACCGCTGAGGACATGGGAAAGAAGTTCTACATTGTCTGGACCAAGCTGGACATGGA
Ppy_IRGM_s   354 ACAATTCAGCATGAATCAGGTGATGCTTGCCAAAACCGCTGAGGACATGGGAAAGAAGTTCTACATTGTCTGGACCAAGCTGGACATGGA
Hga_IRGM     354 ACAATTCAGTATGAATCACGTGATGCTTGCCAAAACCACTGAGGACATGGGAAAGAAGTTCTACGTTGTCTGGACCAAGCTGGACATGGA
Hga_IRGM_s   354 ACAATTCAGTATGAATCACGTGATGCTTGCCAAAACCACTGAGGACATGGGAAAGAAGTTCTACGTTGTCTGGACCAAGCTGGACATGGA
Rh_IRGM      353 ACAATTCAGCATGAATCATGTGATGCTTGCCAAAACCACTGAGGACATGGGAAAGAAGTTCTACATTGTCTGGACCAAGCTGGACATGGA
Pha_IRGM     353 ACAATTCAGCATGAATCATGTGATGCTTGCCAAAACCACTGAGGACATGGGAAAGAAGTTCTACATTGTCTGGACCAAGCTGGACATGGA
Cja_IRGM     354 ACAGTTCAGCATGAATCATGTGATCCTTGCCAAAACCATTGAGGACATGGGAAAGAAGTTCTACATTGTCTGGACCAAGCTGGACATGGA
IRGM9        357 ACAGTTCAGCATGAATCACGTGATGCTTGCCAAAACAGTGGAGGACATGGGAAAACAGTTCTACATTGTCTGGACCAAGCTGGACATGGA
IRGM8        357 ACAGTTCAGCATGAATCACGTGATGCTTGCCAAAACAGTGGAGGACATGGGAAAACACTTCTACATTGTTTGGACCAAGCTGGACATGGA
IRGM7        357 ACAGTTCAGCATGAATCACGTGATACTTGCAAAAACCATGTGGGACATGAGAAAACATTTTTACGTTGTCTGGACCAAGCTGGACATGGA
IRGM5        357 ACAGTTCAGCATGAATCTTGTGAAGCTTGTCAAAGCCATCCAAAGACAGGGGAAAAGGTTCTACATTGTCTGGACCAAGCTGGACAGGGA
IRGM6        357 ACAGTTCAGCATGAATCTTGTGAAGCTTGCCAAGGCCATCCAGGTCCTGGGAAAGAGATTCTACATTGTCTGGACCAAGCTGGACAGGGA
IRGM4        357 ACAGTTCAGCATGAATCTCGTGAAGCTTGTCAAAAGCATCCAGGGACAAGGAAAGAGGTTTTACATCGTCTGGACCAAGTTGGACAGAGA
Irgm2        357 GCAGTTCAGCTTAAATCATGTGAAGCTGGCCATAACCATGCAGAGGATGAGAAAGAGGTTCTATGTCGTCTGGACCAAGCTGGACAGGGA
Irgm3        360 GCAGTTCAGCTCAAATCATGTGAAGCTGGCCATAACCATGCAGAGGATGAGAAAGAGGTTCTATGTCGTCTGGACCAAGCTGGACAGGGA
Irgm1        357 GCAGTTCAGCTCGAATCATGTGAAGCTGTCCAAAATTATCCAGAGCATGGGAAAGAGGTTCTATATTGTCTGGACCAAGCTGGACAGGGA

Hs_IRGM      444 CCTCAGCACAGGTGCCCTCCCAGAAGTGCAGCTACTGCAGAT---CAGAGAAAATGTCCTGGAAAATCTCC
Ptr_IRGM     444 CCTCAGCACAGGTGCCCTCCCAGAAGTGCAGCTACTGCAGAT---CAGAGAAAATGTCCTGGAAAATCTCC
Ggo_IRGM     444 CCTCAGCACAGGTGCCCTCCCAGAAGTGCAGCTACTGCAGAT---CAGAGAAAATGTCCTGGAAAATCTCC
Ppy_IRGM     444 CCTCAGCACAGGTGCCCTCCCAGAAGTGCAGCTACTGCAGAT---CAGAGAAACGTCCTGGAAAATCTCC
Ppy_IRGM_s   444 CCTCAGCACAGGTGCCCTCCCAGAAGTGCAGCTACTGCAGAT---CAGAGAAACGTCCTGGAAAATCTCC
Hga_IRGM     444 CCTCAGCACAGGTGCCCTCCCAGAAGTGCAGCTACTGCAGAT---CAGAGAAAATGTCCTGGAAAATCTCC
Hga_IRGM_s   444 CCTCAGCACAGGTGCCCTCCCAGAAGTGCAGCTACTGCAGAT---CAGAGAAAATGTCCTGGAAAATCTCC
Rh_IRGM      443 CCTCAGCACAGGTGCCCTCCCAGAAGTGCAGCTACTGCGGAT---CAGAGAAAATGTCCTGGAAAATCTCC
Pha_IRGM     443 CCTCAGCACAGGTGCCCTCCCAGAAGGCCAGCTACTGCGGAT---CAGAGAAAATGTCCTGGAAAATCTCC
Cja_IRGM     444 TCTCAGCACAGGTGCCCTCCCAGAAGTGCAGCTACTGTAAAT---CAGAGAAAATGTCCTGGAAAGTCTCC
IRGM9        447 TCTCAACACAAGTGCCCTCCCCAAAGGGCAGCTGGGAAGATTATCAGGGAAAATATTCTGGAAAATCTCC
IRGM8        447 TCTCAACACAAGTGCCCTCCCAAAAGGGCAGCTGCGGCAGATTATCAGGGAAAATATTCTGGAAAATCTCC
IRGM7        447 TCTCAACACAAGTGCCCTCCCTGAAGGGCAGCTGCTGCAGATTATCAGGGAAAATATTCTGGAAAATCTCC
IRGM5        447 CCTCAGCACACGTGTCCTTCCAGAGGAACAAGTCCTGCAGAATATATGGGAGAATATCCAGGAAACTCTCC
IRGM6        447 CCTCAGCACAAGTGCTCTCTTGAAAGAACGTCTCCTGCAGAATATTCAGGAGAATATTCAGGAAAATCTCC
IRGM4        447 CCTCAGTACATGTGTCCTTTCAGAAGAACAACTCCTGCGGAATATCCGGAGAATATCAGGGAAACTCTCC
Irgm2        447 CCTCAGCACAAGTACCTTCCCTGAACCCCAGCTACTGCAGAGTATCCAAAGGAATATCCGGGATAGTCTTC
Irgm3        450 CCTCAGCACAAGTACCTTCCCTGAACCCCAGCTACTGCAGAGTATCCAAAGGAATATCCGGGAGAATCTCC
Irgm1        447 CCTCAGCACCAGTGTCCTATCAGAGGTCCGGCTCCTACAGAATATCCAGGAGAATATCCGAGAGAATCTGC
```

**Supplementary Note Figure 4. Amino acid alignment of the *IRGM* proteins (full length)**

Protein sequences of mouse, dog and prosimian (*M. murinus*) *IRGM* show close homology both in N-terminal GTPase binding domain (G domain) and C terminus. Canonical GTPase motifs are indicated by red boxes. *IRGM7* and *IRGM9* (gray mouse lemur (*M. murinus*) *IRGM* GMS type GTPases). *IRGM8* is considered to be pseudogene and is not included. *IRGM*4, *IRGM*5, *IRGM6* (Dog *IRGM* GMS type GTPases). *Irgm1, Irgm2, Irgm3* (mouse *IRGM* GMS type GTPases).

```
IRGM7     1 ------------------------------------------------------MLSRVEAMNIEKALADGNLPEVVSAI
IRGM9     1 MLSQMTLMHPPNSPALLPLQVPPLLTDVTAFPLSPHTPLSASLTAVLPQCKDWSMLSKVEALSIEKAIAGGNLPELVSAV
IRGM5     1 ---------------------------MTQPNHSLHIPLSTSFTSIVPYNMGWTVLPKATATNIEKALGDGKLLEVVSMI
IRGM6     1 -----------LHCFFPLLQVTPLLSDVTQPTHSLHTPLLTSSNYDMPYNMGWSSLSKETAINIEKALGGRKLLEVVPMV
IRGM4     1 ---------------------------MAQPTQSLHTPSPTSFTSTVPYHKGGSILSESGAMNIEKALGEGKLLDMVSVV
Irgm1     1 --------------MKPSHSSCEAAPLLPNMAETHYAPLSSAFPFVTSYQTGSSRLPEVSRS-TERALREGKLLELVYGI
Irgm2     1 -------------MPT--SRVAPLLDNMEEAVESPEVKEFEYFSDAVFIPKDGNTLSVGVIKRIETAVKEGEVVKVVSIV
Irgm3     1 -------MDLVTKLPQNIWKTFTLFINMANYLKRLISPWSKSMTAGESLYSSQNSSSPEVIEDIGKAVTEGNLQKVIGIV

IRGM7    27 RESLKMASRTPVNVALTGNSGNDMSSFINPLRNIGHEEEASVPVAVLKTTQTRACYLS-PHFPNVVLWDLPGTECAAQSL
IRGM9    81 RETVKMVSRTPVNVAVTGDSGNGMSSFINALRNIGHEEEASAPVGVLKTTQTHACYLS-PHFPNVVLWDLPGTVSAAQSL
IRGM5    54 RETLETVSSAPVSIAVTGDSGNGMSSFINALREIGHDEKDSAPTGVVRTTQVPTCYSS-SHFPYMELWDLPGTGTGTQSL
IRGM6    70 RETLERASSVPLRIAVTGDSGNGMSSFINALRGIGHDEEDSAPTGVVKTTQIPTCYSY-PHFPNVELWDLPGTGAGTQSL
IRGM4    54 RETLETASSVPVSIAVTGDSGNGMSTFINALRKIGHNEEDSAPTGVVRTTQIPTCYSF-SDIPNVELWDLPGTGAATQNL
Irgm1    66 KETVATLSQIPVSIFVTGDSGNGMSSFINALRVIGHDEDASAPTGVVRTTKTRTEYSS-SHFPNVVLWDLPGLGATAQTV
Irgm2    66 KEIIQNVSRNKIKIAVTGDSGNGMSSFINALRLIGHEEKDSAPTGVVRTTQKPTCYFS-SHFPYVELWDLPGLGATAQSV
Irgm3    74 KDEIQSKSRYRVKIAVTGDSGNGMSSFINALRFIGHEEEDSAPTGVVRTTKKPACYSSDSHFPYVELWDLPGLGATAQSV
```

GXXXXGMS

G1

DXXG

G3

```
IRGM7   106 ENYSMEMQFSRYDFFIIIVSEQFSMNHVILAKTMWDMRKHFYVVWTKLDMDLNTSALPEGQLLQIIRENILENLQKQRVC
IRGM9   160 ENYATEMQFSRYDFFIIIASEQFSMNHVMLAKTVEDMGKQFYIVWTKLDMDLNTSALPKGQLGKIIRENILENLQKQRVC
IRGM5   133 ENYLEKIHFSQYDLFIIIASEQFSMNLVKLVKAIQRQGKRFYIVWTKLDRDLSTRVLPEEQVLQNIWENIQETLQKVGVC
IRGM6   149 ENYLEEMKFSWYDLFIIIASEQFSMNLVKLAKAIQVLGKRFYIVWTKLDRDLSTSALLKERLLQNIQENIQENLQKERVF
IRGM4   133 ETYLEEMQFSKYDLFIIIASEQFSMNLVKLVKSIQGQGKRFYIVWTKLDRDLSTCVLSEEQLLRNIRENIRETLHKEGVC
Irgm1   145 EDYVEEMKFSTCDLFIIIASEQFSSNHVKLSKIIQSMGKRFYIVWTKLDRDLSTSVLSEVRLLQNIQENIRENLQKEKVK
Irgm2   145 ESYLEEMQISIYDLIIIVASEQFSLNHVKLAITMQRMRKRFYVVWTKLDRDLSTSTFPEPQLLQSIQRNIRDSLQKEKVK
Irgm3   154 ESYLEEMQISTFDLIIIVASEQFSSNHVKLAITMQRMRKRFYVVWTKLDRDLSTSTFPEPQLLQSIQRNIRENLQQAQVR
```

TKXD

G4

```
IRGM7   186 GPPIFLVSSFDPLSYDFPKLRDSLQMDLMKNRYHELLQNLSHMCERAVNDKVSFLQKK-IATESLQDACG-ISDADDLAA
IRGM9   240 EPPIFLVSSFDPLSYDFPKLRDSLQMDLMKNRRHELLQNLSHTCERAVNDKVSFLQKK-IATESLQDACG-ISDADDLAA
IRGM5   213 EPIIFLVSSFEPLLHDFPELRDALNRDISDIRYCGPLENLSDTCEKIINDKVTSFQEQ-IGSKTFQDILG-IQDEDDLGQ
IRGM6   229 EPIIFLVSSFEPLLHDFPELRNTLNRDISDIRYCGPLKNLSHTYEKVISDKVTMFRGK-IASKSF-DTLG-IWNADDLGE
IRGM4   213 EPIIFLVSSFNPFLHDFPELRKSLHRDISNIGYRGHLENLTHTCEKVINGKVTTLQGQ-IGSKSFQDILG-IQNANDLGE
Irgm1   225 YPPVFLVSSLDPLLYDFPKLRDTLHKDLSNIRCCEPLKTLYGTYEKIVGDKVAVWKQR-IANESLKNSLG-VRDDDNMGE
Irgm2   225 EHPMFLVSVFKPESHDFPKLRETLQKDLPVIKYHGLVETLYQVCEKTVNERVESIKKS-IDEDNLHTEFG-ISDPGNAIE
Irgm3   234 DPPLFLISCFSPSFHDFPELRNTLQKDIFSIRYRDPLEIISQVCDKCISNKAFSLKEDQMLMKDLEAAVSSEDDTANLER

IRGM7   264 CLKAYQLLFGVDDESLWQVAQRVGRTFADYTNITKSCDVQGLSTRNWKLTCMTCTVFRAFLGLLRCIPWLGNLTIHYFRR
IRGM9   318 CLKAYQLLFGVDDESLWQVAQRVGRTFADYTNITKSCDVQGLSTRNWKLTCMTCTVFRAFLGLLRCIPWLGNLIIGYFRG
IRGM5   291 CLIAYHLFFGVDDKSLQQMAQSMGKPMEEYRAIMKSQDVHTVLTGDWALSCMNCKTASYLYSILSYIPFLGDTVINYLRV
IRGM6   306 CLIAYHLFFGVDDESLQQIAQSMGKPMEEYRAIMKSRDLHTIIRGDWAVSCMNCNTSSCLYTILRYIPLLGDFIINFLRK
IRGM4   291 FLNAYHRLFGVDDDSLQEVAQSMGKPKEEYKAIMKSQDLHTALAWDWALSWMNCNAASYLYSVLSYIPILGTTGIHYLKW
Irgm1   303 CLKVYRLIFGVDDESVQQVAQSMGTVVMEYKDNMKSQNFYTLRREDWKLRLMTCAIVNAFFRLLRFLPCV----CCCLRR
Irgm2   303 IRKAFQKTFGLDDISLHLVALEMKNKHFNT-SMESQETQRYQQ-DDWVLARLYRTGTRVGSIGFDYMKCC--FTSHHSRC
Irgm3   314 GLQTYQKLFGVDDGSLQQVARSTGRLEMGSRALQFQDLIKMDRRLELMMCFAVNKFLRLLESSWWYGLWN--VVTRYFRH

IRGM7   344 KKQRCLLEIVAEDTKAILRKVLEDSIIPQ---
IRGM9   398 KKQRCLLEIVAEDTKAIIRKVLEDSIIPQ---
IRGM5   371 WKHRHFLEIVAKDTRSIVKKILTDSII-----
IRGM6   386 WKHRRLLEIVAEDTRTILKKILKDSII-----
IRGM4   371 WSQGHLLEIVAEDTKTILKKILEDAII-----
Irgm1   379 LRHKRMLFLVAQDTKNILEKILRDSIFPPQI-
Irgm2   379 KQQKDILDETAAKAKEVLLKILRLSIPHP---
Irgm3   392 QRHKLVIEIVAENTKTSLRKALKDSVLPPEIH
```

**Supplementary Note Figure 5. Nucleotide alignment of Old World monkey *IRGM* genes**

Red highlighted sequence indicates the position of either a frameshift mutation or an in-frame stop codon. Species names are indicated as: Hs (*Homo sapiens*), Rh (rhesus macaque, *Macaca mulatta*), Cja (*Callithrix jacchus*), (Mar) *Macaca arctoides*, (Mni) *Macaca nigra*, (Mmu), (Mfa) *Macaca fascicularis*, (Pan) *Papio hamadryas anubis*, (Pha) Baboon (*Papio hamadryas hamadryas*), (Cce) *Cercopithecus cephus*, (Cae) *Cercopithecus aethiops*, (Pcr) *Precybitis cristata*, (Cpo) *Colobus polykomos*, (Cgu) *Colobus guereza*.

```
Hs_IRGM      1 GAGACTCTGAAGATAGTGTCCAGGACACCAGTTAACATCACTATGGCAGGGGACTCTGGCAATGGGATGT
Mfa_IRGM     1 GAGACTCTGAAGATAGTGTCCAGGACACCAGTCAACATTGCTATGGCAGGG-ACTCTGGCAGTGGGATGA
Rh_IRGM      1 GAGACTCTGAAGATAGTGTCCAGGACACCAGTCAACATTGCTATGGCAGGG-ACTCTGGCAGTGGGATGA
Mni_IRGM     1 GAGACTCTGAAGATAGTGTCCAGGACACCAGTCAACATTGCTATGGCAGGG-ACTCTGGCAGTGGGATGA
Mar_IRGM     1 GAGACTCTGAAGATAGTGTCCAGGACACCAGTCAACATTGCTATGGCAGGG-ACTCTGGCAGTGGGATGA
Pan_IRGM     1 GAGACTCTGAAGATAGTGTCCAGGACACCAGTCAACATTGCTATGGCAGGG-ACTCTGGCAGTGGGATGA
Pha_IRGM     1 GAGACTCTGAAGATAGTGTCCAGGACACCAGTCAACATTGCTATGGCAGGG-ACTCTGGCAGTGGGATGA
Cce_IRGM     1 GAGACTCTGA-GATAGTGTCCAGGACACCAGTCAACATTGCTATGGCAGGG-ACTCTGGCAGTGGGATGA
Cae_IRGM     1 GAGACTCTGA-GATAGTGTCCAGGACACCAGTCAACATTGCTATGGCAGGG-ACTCTGGCAGTGGCTGA
Cpo_IRGM     1 GAGACTCTGA-GATAGTGTCCAGGACACCAGTCAACATTGCTATGGCAGGG-ACTCTGGCAATGGGATGA
Cgu_IRGM     1 GAGACTCTGAAGATAGTGTCCAGGACACCAGTCAACATTGCTATGGCAGGG-ACTCTGGCAATGGGATGA
Pcr_IRGM     1 GAGACTCTGAAGATAGTGTCCAGGACACCAGTCAACATTGCTATGGCAGGG-ACTCTGGCAATGGGACGA

Hs_IRGM     71 CCACCTTCATCAGTGCCCTTCGAAACACAGGACATGAGGGTAAGGCCTCACCTCCTACTGAGCTGGTAAA
Mfa_IRGM    70 ACACCTTCATCAGTGCCCTTTGAAACACAGGACATGAGGGGAAGCCCTCACCTCCTACTGGGCTGGTAAA
Rh_IRGM     70 ACACCTTCATCAGTGCCCTTTGAAACACAGGACATGAGGGGAAGCCCTCACCTCCTACTGGGCTGGTAAA
Mni_IRGM    70 ACACCTTCATCAGTGCCCTTTGAAACACAGGACATGAGGGGAAGCCCTCACCTCCTACTGGGCTGGTAAA
Mar_IRGM    70 ACACCTTCATCAGTGCCCTTTGAAACACAGGACATGAGGGGAAGCCCTCACCTCCTACTGGGCTGGTAAA
Pan_IRGM    70 ACACCTTCATCAGTGCCCTTTGAAACACAGGACATGAGGGGAAGCCCTCACCTCCTACTGGGCTGGTAAA
Pha_IRGM    70 ACACCTTCATCAGTGCCCTTTGAAACACAGGACATGAGGGGAAGCCCTCACCTCCTACTGGGCTGGTAAA
Cce_IRGM    69 ACACCTTCATCAGTGCCCTTTGAAACACAGGACATGAGGGGAAGGCCTCATCTCCTACTGGGCTGGTAAA
Cae_IRGM    69 ACACCTTCATCAGTGCCCTTTGAAACACAGGACATGAGGGGAAGGCCTCACCTCCTACTGGGCTGGTAAA
Cpo_IRGM    69 ACATCTTCATCAGTGCCCTTTGAAACACAGGACATGAGGGGAAGGCCTCACCTCCTACTGGGCTGGTAAA
Cgu_IRGM    70 ACATCTTCATCAGTGCCCTTTGAAACACAGGACATGAGGGGAAGGCCTCACCTCCTACTGGGCTGGTAAA
Pcr_IRGM    70 ACATCTTCATCAGTGCCCTTTGAAACACAGGACATGAGGGGAAGGCCTCACCTCCTAATGGGCTGGTAAA
```

**Supplementary Note Figure 6. Nucleotide alignment of New World monkey *IRGM* genes**
Red highlighted sequence indicates the position of either a frameshift mutation or an in-frame stop codon. Species names are indicated as: Hs (*Homo sapiens*), (Sbo) *Saimiri boliviensis*, (Cge) Marmoset (*Callithrix geofroyi*), (Cmo) *Callicebus moloch*, (Ppi) *Pithecia pithecia*.

```
Sbo_IRGM    1 AGAGGTGTTCTCTGCCATCAAGGAGACTTTGAAGATAGTGTTCAGGACACCAGTCAACATCGCTATGGCA
Cge_IRGM    1 AGAGGTGGTCTCTGCCATCAAGGAGAGTTTGAAGATAGTGTTCAGGACACCAGTCAACATCGCTATGGCA
Cmo_IRGM    1 AGAGGTGGTCTCTGCCATCAAGGAGACTTTGAAGATAGTGTTCAGGACACCAGTCAACATCGCTATGGCA
Ppi_IRGM    1 AGAGGTGGTCTCTGCCATCAAGGAGACTTTGAAGACAGTGTTCAGGACACCAGTCAACATCGTTATGGCA
Hs_IRGM     1 AGAGGTGATCTCTAACATCAAGGAGACTCTGAAGATAGTGTCCAGGACACCAGTTAACATCACTATGGCA

Sbo_IRGM   71 GGGGACTCTAGCAATAGCATGTCCACCTTCATCAGTGCACTGCAAAACACAGGGCATGAG---------C
Cge_IRGM   71 GGGGACTCTGGCAATAGCATATCCACCTTCATCAGTGCACTTCAAATCGCAGGGCATGAGGCGAAGGCCT
Cmo_IRGM   71 GGGGACTCTGGCAATAGCATGTCCACCTTCATCAGTGCACTTCAAAACACAGGGCATGAGGGGAAGGCCT
Ppi_IRGM   71 GGGGACTCTGGCAATAGCATGTCCACCTTCATCAGTGCCCTTCAAAACACAGGGCATGAGGGGAAGGCCT
Hs_IRGM    71 GGGGACTCTGGCAATGGGATGTCCACCTTCATCAGTGCCCTTCGAAACACAGGACATGAGGGTAAGGCCT

Sbo_IRGM  132 TTTTACCTACTAGGCTGGTAAAAGCTACCCAAAGATGTGCCT---ATTTCTCTTCCCACTTTCCAAATGT
Cge_IRGM  141 CACCTCCTACTGGGCTGGTAAAAGCTACCCAAAGATGTGCCTCCTATTTCTCTTCCCGCTTTCCAAATGT
Cmo_IRGM  141 CACCTCCTACTGGGCTGGTAAA-GCTACCCAAAGATGTGCCTCCTATTTCTCTTCCCACTTGCCAAATGT
Ppi_IRGM  141 CACCTCCTACTGGGCTGGTAAAAGCTACGCAAAGATGTGCCTCTTATTTCTCTTCCCACTTTCCAAATGT
Hs_IRGM   141 CACCTCCTACTGAGCTGGTAAAAGCTACCCAAAGATGTGCCTCCTATTTCTCTTCCCACTTTTCAAATGT

Sbo_IRGM  199 GGTGCTGTGGGACCTGCCTGGTGCAGGGTCTGCCACCAAAACTCTGGAGAACTACCTGACGGAAATGTAG
Cge_IRGM  211 GGTGCTGTGGGATCTGCCTGGAGCAGGGTCTGCCACCAAAACTCTGGAGAACTACCTGATGGAAATGTAG
Cmo_IRGM  210 GGTGCTGTGGGACCTGCCTGGCACAGGGTCTGCCACCAAAACTCTGGAGAACTACCTGATGGAAATGTAG
Ppi_IRGM  211 GGTGCTGTGGGACCTGCCTGGCACAGGGTCTGCCACCAAAACTCTGGAGAACTACCTGATGGAAATGTAG
Hs_IRGM   211 GGTGTTGTGGGACCTGCCTGGCACAGGGTCTGCCACCACAACCCTGGAGAACTACCTGATGGAAATGCAG

Sbo_IRGM  269 TTCAGCCAGTGTGACTTCATCATGGTTGCATCTGCACAATTCAGCATGAATCACGT-GATCCTTGCCAAA
Cge_IRGM  281 TTCAACCAATATGACTTCATCATGGTTGCATCTGCACAGTTCAGCATGAATCATGT-GATCCTTGCCAAA
Cmo_IRGM  280 TTCAACCAGTATGACATCATCATGGTTGCATCTGCACAACTCAGCATGAATCATGTTGATGCTTGCCAAA
Ppi_IRGM  281 TTCAACCAGTATGACTTCATCGTGGTTGCATCTGCACAATTCAGCATGAATCATGTTCATGCTTGCCAAA
Hs_IRGM   281 TTCAACCGGTATGACTTCATCATGGTTGCATCTGCACAATTCAGCATGAATCATGT-GATGCTTGCCAAA

Sbo_IRGM  338 ACCATTGAGGACATGGGAAAGAAGTTCTACATTGTCTGGACCAAGCTGGACATGGATCTCAGCACAGGTG
Cge_IRGM  350 ACCATTGAGGACATGGGAAAGAAGTTCTACATTGTCTGGACCAAGCTGGACATGGATCTCAGCACAGGTG
Cmo_IRGM  350 ACCATTGAGGACATGGGAAAGAAGTTCTACATTGTCTGGACCAAGCTGGACATGGATCTCAGCACAGGTG
Ppi_IRGM  351 ACCATTGAGGACGTGGGAAAGAAGTTCTGCATTGTCTGGACCAAGCTGGACATGGATGTCAGCACAGGTG
Hs_IRGM   350 ACCGCTGAGGACATGGGAAAGAAGTTCTACATTGTCTGGACCAAGCTAGACATGGACCTCAGCACAGGTG

Sbo_IRGM  408 CCCTCCCAGAAGTGCAGCTACTGTAAATCAGAGAAAATGTCC
Cge_IRGM  420 CCCTCCCAGAAGTGCAGCTACTGTAAATCAGAGAAAATGTCC
Cmo_IRGM  420 CC-TCTCAGAAGTGCAGCTACTGTAAATCAGAGAAAATGTCC
Ppi_IRGM  421 CCCTCCCAGAAGTGCAGCTACTGTAAATCAGAGAAAATGTCC
Hs_IRGM   420 CCCTCCCAGAAGTGCAGCTACTGCAGATCAGAGAAAATGTCC
```

**Supplementary Note Table 4. *Microcebus murinus* BAC clones**

An *IRGM9* PCR product generated from *M. murinus* genomic DNA was used as a probe to screen a *M. murinus* BAC library (CHORI-257). Clones were tested for *IRGM* content by direct sequencing of the BAC and by sequencing of PCR amplified product. Depending on the approach, the results revealed single or multiple copies of *IRGM*.

| Clone ID | Direct BAC sequencing | PCR | *IRGM* |
|---|---|---|---|
| 190D1 | (+) multiple | (+) single | ORF |
| 174K21 | (+) multiple | (+) single | ORF |
| 243N23 | (+) multiple | (+) single | ORF |
| 387J22 | (+) single | (+) single | ORF |
| 466C7 | (+) single | (+) single | ORF |
| 110K8 | (+) multiple | (+) multiple | ORF |
| 295F3 | (+) single | (+) single | ORF |
| 195B7 | (+) single | (+) multiple | ORF |
| 231F17 | (+) single | (+) single | ORF |
| 324L12 | | (+) multiple | ORF |
| 197G14 | | (+) single | ORF |
| 461N16 | | (+) single | stop codon |
| 482L2 | | (+) multiple | ORF |
| 322D19 | | (+) single | stop codon |
| 310H1 | | (+) single | ORF |
| 197i14 | (+) single | (+) single | ORF |
| 233o12 | | (+) single | ORF |
| 325M3 | (+) single | (+) multiple | ORF |

**Supplementary Note Table 5. *Lemur catta* BAC library and FISH**
An *IRGM9* PCR product generated from *M. murinus* genomic DNA was used as a probe to screen a ringtailed lemur genomic BAC library (LB-2). FISH was performed with eight clones and six of the eight mapped to the same region syntenic with human chromosome 5 (*map location of human IRGM*). Analysis of interphase nuclei (Figure 2 main text) showed the presence of multiple signals consistent with a tandem gene family. One clone, 61D22, generated multiple pericentromeric signals.*Data according to Cardone et al., (2002)[12]

| Clone IDs | LCA FISH mapping | Orthologous regions in Hs* |
|---|---|---|
| LB2-61D22 | 4q, 10qcen,12qcen,21q | 5q31-qter, 12pter-q24,10q and 22qter/12qter |
| LB2-61A22 | 4q | 5q31-qter |
| LB2-77B23 | 4q | 5q31-qter |
| LB2-77A24 | 4q | 5q31-qter |
| LB2-138G6 | 4q | 5q31-qter |
| LB2-191B24 | 4q | 5q31-qter |
| LB2-217I21 | 3q | 1pter-q23 |
| LB2-277N18 | 2pcen | 16p |

**Supplementary Note Table 6. Primate DNA sample IDs and genotyping results**
The *IRGM* locus was sequenced in multiple individuals from various anthropoid species. Table provides information on the sample IDs and the number of stop codons that were observed in the ORF based on direct resequencing of the PCR product.

| Common Name | Species | Name or ID | Sex | Stop Codons |
|---|---|---|---|---|
| Human | *Homo sapiens* | GM15510 | F | 0 |
| Human | *Homo sapiens* | Czech GM 15724 | M | 0 |
| Human | *Homo sapiens* | ND05418 | M | 0 |
| Human | *Homo sapiens* | ND05586 | M | 0 |
| Human | *Homo sapiens* | CEPH 11840 | N/A | 0 |
| Chimpanzee | *Pan troglodytes* | Clint | M | 0 |
| Chimpanzee | *Pan troglodytes* | Katie | F | 0 |
| Chimpanzee | *Pan troglodytes* | Logan | M | 0 |
| Chimpanzee | *Pan troglodytes* | PR00238 | M | 0 |
| Chimpanzee | *Pan troglodytes* | PR00052 | F | 0 |
| Gorilla | *Gorilla gorilla* | Bahati | F | 0 |
| Gorilla | *Gorilla gorilla* | Rollie | M | 0 |
| Gorilla | *Gorilla gorilla* | Kwan | M | 0 |
| Orangutan | *Pongo pygmaeus* | PUTI (EEE0002PPY) | M | 0 |
| Orangutan | *Pongo pygmaeus* | HATI (EEE0003PPY) | F | 1* |
| Orangutan | *Pongo pygmaeus* | TENGKU (EEE0004PPY) | M | 0 |
| Orangutan | *Pongo pygmaeus* | AG12256 | F | 1* |
| Orangutan | *Pongo pygmaeus* | AG05252 | M | 0 |
| Orangutan | *Pongo pygmaeus* | AG06105 | F | 0 |
| Orangutan | *Pongo pygmaeus* | Susie (PR01109) | F | 1* |
| Orangutan | *Pongo pygmaeus* | PPY-9 | F | 0 |
| Gibbon | *Hylobates pileatus* | PR00243 | F | 0 |
| Gibbon | *Hylobates gabriellae* | PR00652 | F | 1* |
| Colobus | *Colobus guereza* | 7029-2192 | M | 2 |
| Colobus | *Colobus polykomos* | LA16I | N/A | 2 |
| Presbytis | *Presbytis cristata* | N/A | N/A | 2 |
| Cercopithecus | *Cercopithecus aethiops* | N/A | N/A | 2 |
| Cercopithecus | *Cercopithecus cephus* | LA38 | N/A | 2 |
| Baboon | *Papio hamadryas anubis* | SFBR-6738 | F | 2 |
| Baboon | *Papio hamadryas anubis* | SFBR-17260 | F | 2 |
| Baboon | *Papio hamadryas hamadryas* | SFBR-8320 | M | 2 |
| Baboon | *Papio hamadryas hamadryas* | SFBR-2X0236 | F | 2 |
| Baboon | *Papio hamadryas hamadryas* | SFBR-2X0331 | F | 2 |
| Macaque | *Macaca mulatta* | # 17748 | N/A | 2 |
| Macaque | *Macaca mulatta* | #17753 | N/A | 2 |
| Macaque | *Macaca mulatta* | # 17756 | N/A | 2 |
| Macaque | *Macaca mulatta* | # 18398 | N/A | 2 |

| | | | | |
|---|---|---|---|---|
| Macaque | *Macaca mulatta* | # 18404 | N/A | 2 |
| Macaque | *Macaca fascicularis* | Mariano | N/A | 2 |
| Macaque | *Macaca nigra* | Mariano | N/A | 2 |
| Macaque | *Macaca arctoides* | Mariano | | 2 |
| Marmoset | *Callithrix geofroyi* | PR01094 | F | 2 |
| Pithecia | *Pithecia pithecia* | PR00239 | M | 2 |
| Callicebus | *Callicebus moloch* | PR00742 | F | 2 |
| Saimiri | *Saimiri boliviensis* | PR00474 | M | 2 |

**individual was heterozygous for stop codon (CGA/TGA).*

**Supplementary Note Table 7. PCR assays**

PCR was performed in 20 µl reactions composed of 0.8 µl of a 10 µM dilution of the forward primer and reverse primer, 10 µl of Roche (11636103001) PCR Master Mix. The following PCR conditions were used: 1 min at 94°C, followed by 45 cycles at 94°C for 30 sec, 55°C 30 sec, and 72°C for 30 sec followed by 7 min at 72°C. PCR conditions for (A) and (B) are the same except that (A) is five cycles more than (B). The following real-time PCR conditions (C) were used: 3 min at 95°C, followed by 50 cycles at 95°C for 15 sec, 55°C 20 sec, and 72°C for 20 sec.

| Primer1 | Sequence | Primer2 | Sequence2 | Length | PCR Conditions |
|---|---|---|---|---|---|
| **RT-PCR** | | | | | |
| 5utr(Rh)-F | TCAAAGGCTGGTGGCTTACTTTGTA | 5utr(Rh)-R | AAGGGTTTAGGATGCAGCTAATGGA | 144 | A |
| 5utr(Hs)-F | TCTCCTCCTCTCCCTCACTTCAGTT | 5utr(Hs)-R | GCACTTGGGACACTCTGTCGTATCT | 181 | A |
| 5utr(Ptr)-F | TCTCCTCCTCTCCCTCACTTCAGTT | 5utr(Ptr)-R | GCACTTGGGACACTCTGTCGTATCT | 181 | B |
| IRGM_Cja_F | AATGTTGAGAGAGCCTCAGCAGAT | IRGM_Cja_R | GGAGACTTTCCAGGACATTTTCTCT | 507 | B |
| IRGM_Rh_F | TGAGAAAGCCTTAGTAGGTGGGAAC | IRGM_Rh_R | GGAGATTTTCCAGGACATTTTCTCT | 502 | B |
| IRGM_Hs_F | CAGGACACCAGTTAACATCACTATG | IRGM_Hs_R | GATTTTCCAGGACATTTTCTCTGAT | 428 | B |
| IRGMMmu-F1 | ATGAGCATTGAGAAAGCCATAGCAG | IRGMMmu-R1 | CAAACAGCAATTGGTAGGCTTTCAG | 795 | B |
| GAPDH-F | ATGACAACTTTGGTATCGTGGAAGG | GAPDH-R | GAAATGAGCTTGACAAAGTGGTCGT | 442 | B |
| UBE1-F | GAAGATCATCCCAGCCATTG | UBE1-R | TTGAGGGTCATCTCCTCACC | 255 | B |
| **Real-Time PCR** | | | | | |
| IRGM(b)-F | CACAACCCTGGAGAACTACCTGATG | IRGM(b)-R | CAGGCCAGCCGCTCCTTCTG | 246 | C |
| IRGM(c)-F | AAGCTAGACATGGACCTCAGCACAG | IRGM(c)-R | TGGTTTGGGTACACTTGTTGGTTTC | 125 | C |
| IRGM(d)-F | GCAGATCAGAGAAAATGTCCTGGAA | IRGM(d)-R | GGGGACAGTTTCCTTCCATCATAAG | 211 | C |
| IRGM(all)-F | GCAGATCAGAGAAAATGTCCTGGAA | IRGM(all)-R1 | TGGCTAGCTGTTGAATATCCTGAGC | Multiple products | C |
| | | IRGM(all)-R2 | ATTTCTGGGCCTTGTGGAATTCTCT | | |
| **5'RACE PCR** | | | | | |
| 5' Anc | GGCCACGCGTCGACTAGTACGGGIIGGGIIGGGII | IRGM-rGMS | ATATTTCTGGGCCTTGTGGAATTCTC | 1706 | B |
| UAP | $(CUG)_4$GGCCACGCGTCGACTAGTAC | IRGMr1 | GATTTTCCAGGACATTTTCTCTGAT | 1630 | B |
